# Engineering Phage Nanocarriers Integrated with Bio‐Intelligent Plasmids for Personalized and Tunable Enzyme Delivery to Enhance Chemodynamic Therapy

**DOI:** 10.1002/advs.202308349

**Published:** 2024-04-06

**Authors:** Xiao‐Lin Hou, Bin Zhang, Kai Cheng, Fang Zhang, Xiao‐Ting Xie, Wei Chen, Lin‐Fang Tan, Jin‐Xuan Fan, Bo Liu, Qiu‐Ran Xu

**Affiliations:** ^1^ Britton Chance Center for Biomedical Photonics at Wuhan National Laboratory for Optoelectronics – Hubei Bioinformatics & Molecular Imaging Key Laboratory Department of Biomedical Engineering College of Life Science and Technology Huazhong University of Science and Technology Wuhan Hubei 430074 P. R. China; ^2^ Key Laboratory of Biomedical Photonics (HUST) Ministry of Education Huazhong University of Science and Technology Wuhan Hubei 430074 P. R. China; ^3^ NMPA Research Base of Regulatory Science for Medical Devices & Institute of Regulatory Science for Medical Devices Huazhong University of Science and Technology Wuhan Hubei 430074 P. R. China; ^4^ Zhejiang Key Laboratory of Tumor Molecular Diagnosis and Individualized Medicine Zhejiang Provincial People's Hospital Affiliated People's Hospital Hangzhou Medical College Hangzhou Zhejiang 310014 P. R. China

**Keywords:** antioxidant braking, chemodynamic therapy, fenton reaction, Nrf2 signal pathway, phage display technology

## Abstract

Customizable and number‐tunable enzyme delivery nanocarriers will be useful in tumor therapy. Herein, a phage vehicle, T4‐Lox‐DNA‐Fe (TLDF), which adeptly modulates enzyme numbers using phage display technology to remodel the tumor microenvironment (TME) is presented. Regarding the demand for lactic acid in tumors, each phage is engineered to display 720 lactate oxidase (Lox), contributing to the depletion of lactic acid to restructure the tumor's energy metabolism. The phage vehicle incorporated dextran iron (Fe) with Fenton reaction capabilities. H_2_O_2_ is generated through the Lox catalytic reaction, amplifying the H_2_O_2_ supply for dextran iron‐based chemodynamic therapy (CDT). Drawing inspiration from the erythropoietin (EPO) biosynthetic process, an EPO enhancer is constructed to impart the EPO‐Keap1 plasmid (DNA) with tumor hypoxia‐activated functionality, disrupting the redox homeostasis of the TME. Lox consumes local oxygen, and positive feedback between the Lox and the plasmid promotes the expression of kelch ECH Associated Protein 1 (Keap1). Consequently, the downregulation of the antioxidant transcription factor Nrf2, in synergy with CDT, amplifies the oxidative killing effect, leading to tumor suppression of up to 78%. This study seamlessly integrates adaptable T4 phage vehicles with bio‐intelligent plasmids, presenting a promising approach for tumor therapy.

## Graphical abstract

A Fenton reaction function phage vehicle TLDF that flexibly modulates enzyme numbers to remodel the TME. Lox molecules deplete lactic acid, reorganizing the tumor's metabolism and producing H_2_O_2_ to boost dextran iron‐based CDT. Lox consumes oxygen and positive feedback between the enzyme and EPO‐Keap1 plasmid, which synergizes with CDT to enhance the oxidative killing effect.

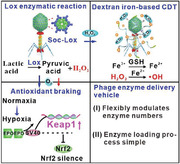

## 1 Introduction

In 2018, the Nobel Prize in Chemistry recognized phage display technology for its pioneering contributions to protein engineering and drug advancement.^[^ ^1^ ^]^ Phage display technology stands as a powerful tool that facilitates the display of exogenous proteins on the phage surface.^[^ ^2^ ^]^ Notably, T4 phage exhibits a substantial protein binding capacity with 1025 display sites, rendering them exceptional vehicles for protein transport.^[^ ^2a^ ^]^ In addition, exogenous proteins can assemble on T4 capsids with remarkable structural and functional integrity.^[^ ^3^ ^]^ The architecture of T4 is decorated with two non‐essential proteins: a small outer capsid protein (Soc, 870 copies/head) and a highly antigenic capsid protein (Hoc, 155 copies/head).^[^ ^4^ ^]^ The N and C termini of Soc are prominently exposed, enabling Soc to function as an adapter for displaying foreign proteins.^[^ ^5^ ^]^ The freely assembled Soc on the phage head demonstrates nanomolar affinity, streamlining the protein loading process and saving time.^[^ ^5^ ^]^ Crucially, the concentration of fusion protein assembly modulates the number of proteins on the phage surface.^[^ ^2a^ ^]^ This tunable feature empowers researchers to adjust protein abundance to meet diverse experimental requirements and applications.

Enzymes catalyzed specific chemical reactions precisely within organisms, effectively transforming endogenous substrates into toxic substances,^[^ ^6^ ^]^ and were widely applied in chemodynamic therapy (CDT).^[^ ^7^ ^]^ As an emerging therapeutic approach, CDT has achieved precise anti‐tumor effects.^[^ ^8^ ^]^ Nevertheless, the depletion of endogenous H_2_O_2_ limited the effectiveness of CDT.^[^ ^9^ ^]^ In contrast to normal tissues, lactic acid, as a crucial energy source, is abundant in the tumor microenvironment (TME) with concentrations (5‐20 µmol/g) surpassing those of glucose (1‐2 µmol/g).^[^ ^10^ ^]^ Mounting evidence suggested that tumor cells derived energy from lactic acid, which played a pivotal role in tumor growth, metastasis, and recurrence.^[^ ^11^ ^]^

A strategy for depleting lactic acid to remodel the TME and achieve continuous catalytic H_2_O_2_ production will significantly enhance the effectiveness of CDT.^[^ ^12^ ^]^ Additionally, the highly reductive TME also hindered the therapeutic effect of CDT.^[^ ^13^ ^]^ The active metabolic activity of tumor cells has many sources of reactive oxygen species (ROS),^[^ ^14^ ^]^ however, nuclear factor E2‐related factor 2 (Nrf2) upregulates metabolic programs to detoxify ROS as a compensatory mechanism.^[^ ^15^ ^]^ In healthy tissues, kelch ECH Associated Protein 1 (Keap1) regulates Nrf2 activity,^[^ ^16^ ^]^ whereas the gain of Nrf2 or loss of the Keap1 function is prevalent in tumor tissues.^[^ ^17^ ^]^ The Nrf2 signaling pathway scavenges ROS‐induced oxidative stress, thereby limiting the applicability of CDT.^[^ ^15^ ^]^ TME‐responsive nanomaterials find extensive application in tumor therapy.^[^ ^17b^ ^]^ For example, hypoxia‐sensitive drugs, such as Tirapazamine, have been developed to precisely target tumor cells.^[^ ^18^ ^]^ Consequently, the hypoxia‐activated smart regulatory plasmid disrupting tumor redox homeostasis is highly prospective for augmenting therapeutic efficacy.^[^ ^13^ ^]^

**Scheme 1:**
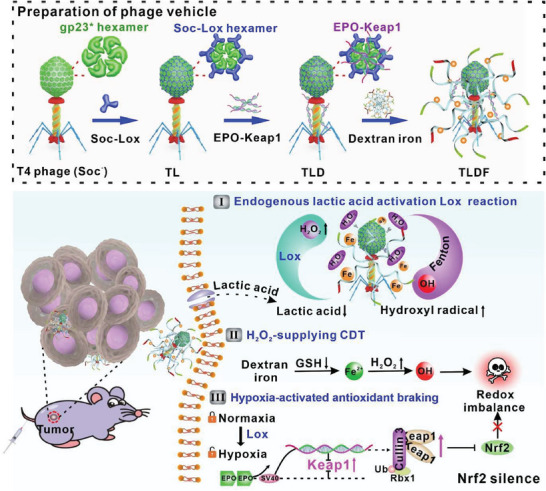
Schematic presentation of the dual‐key activated TLDF probe to disrupt tumor redox homeostasis for enhanced CDT.

Drawing inspiration from the advantages of phage display technology, we introduced an in vitro phage display technology to construct a T4‐Lox‐DNA‐Fe (TLDF) protein vehicle for enhanced CDT (**Scheme** **1**). Regarding the elevated demand for lactic acid in tumors, we strategically presented a customized Soc–Lox fusion enzyme, with Soc serving as an aptamer, displayed on the T4 phage surface (T4‐Lox, TL). The molar ratio of the fusion protein and the binding site can be adjusted for flexible regulation of protein abundance. Compared to Hoc, both the N‐ and C‐termini of Soc are well exposed, allowing for flexible adjustment of enzyme position and conformation. Dextran iron, a drug for iron‐deficiency anemia, camouflaged and imparted phage vehicles with a Fenton catalytic function. Utilizing endogenous lactic acid as a “key”, the Lox enzymatic process was activated, remodeling the tumor's energy metabolism and generating H_2_O_2_ to enhance dextran iron‐based CDT. Drawing inspiration from the erythropoietin (EPO) biosynthetic process, we incorporated a double‐tandem EPO enhancer into the EPO‐Keap1 plasmid, conferring it with hypoxia activation capacity. This will enhance the controllability of gene therapy and downregulated Nrf2. In vitro and in vivo results demonstrated that the two‐pronged strategy of H_2_O_2_‐supplying CDT and gene‐regulated antioxidant brakes disrupted redox homeostasis, resulting in mitochondrial dysfunction in tumor cells. This study introduced a tunable enzyme vehicle through phage display technology in conjunction with bio‐intelligent plasmids to enhance CDT, thus unveiling a novel anti‐tumor strategy.

## 2 Results and Discussion

### 2.1 Preparation and Characterization of TLDF

**Figure 1:**
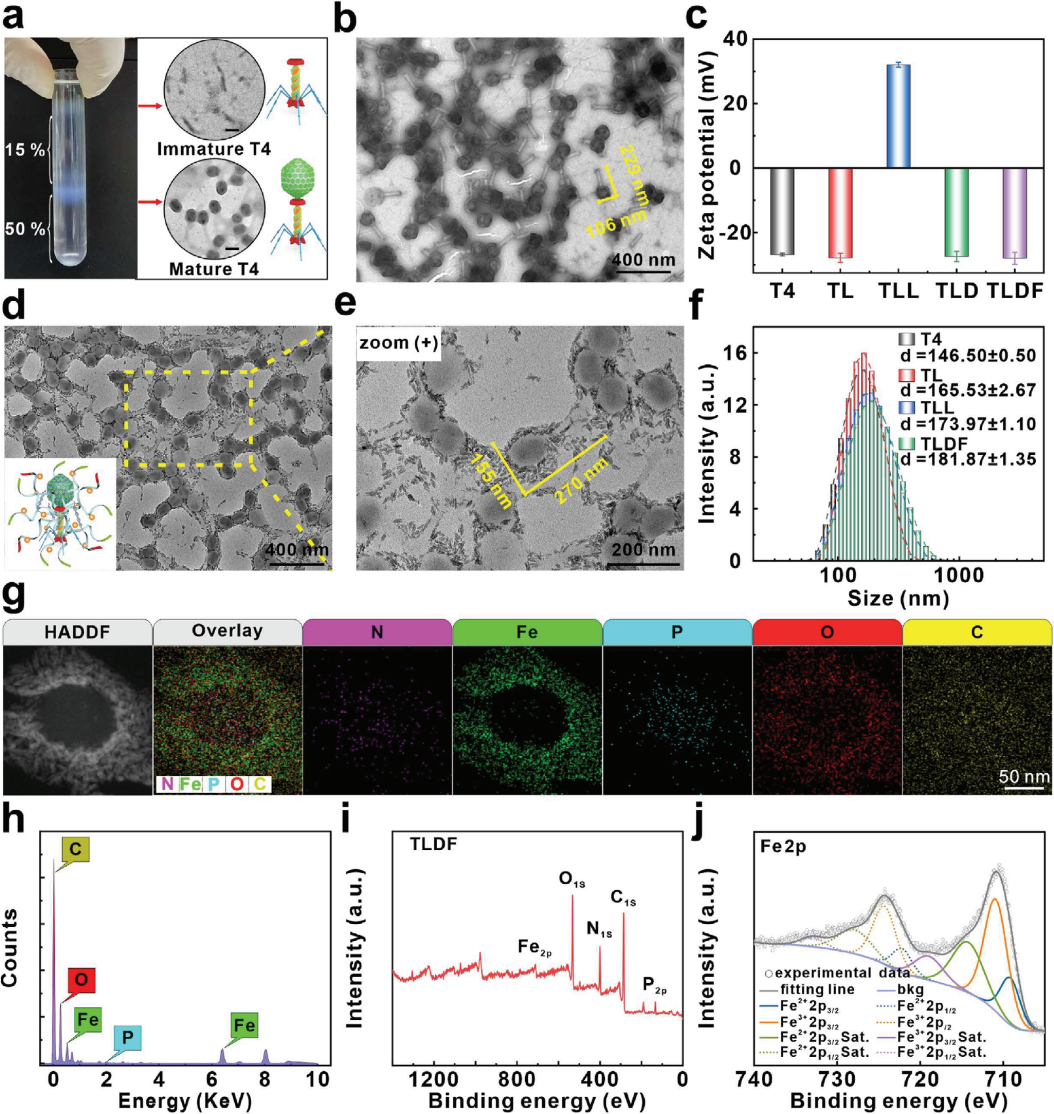
Preparation and characterization of TLDF vehicle. a) Purification of T4 phage by sucrose density gradient centrifugation and corresponding phage TEM images (Scale bar: 100 nm). b) TEM images of T4 and d,e) TLDF. c) Zeta potentials of T4, TL, TLL, TLD, and TLDF and f) corresponding hydrodynamic diameter (*n* = 4). g) HAADF‐STEM image of TLDF and elemental mapping images. h) Energy dispersive spectra TLDF. i), j) XPS spectrum of TLDF and high‐resolution scanning energy spectra of iron.

For the construction of an enzyme delivery system, T4 phage with the Soc capsid gene deletion was employed as a nanocarrier (T4^△Soc^ denoted as T4). The deletion of the Soc capsid protein did not hinder the recognition and infiltration of T4 into the *Escherichia coli* (*E. coli*) BL21 strain (Figure S1, Supporting Information). The amplified T4 underwent purification through sucrose density gradient centrifugation to eliminate impurities, including immature phages and bacterial debris (**Figure** **1**a). The results of transmission electron microscopy (TEM) revealed that unassembled tail tubes of T4 were predominantly concentrated in sucrose with densities below 15%, while mature T4 was observed between 15% and 50% (Figure 1a). The matured T4 phage was ≈229 nm long and 106 nm wide (Figure 1b) and the plaque‐forming unit (pfu) was determined by the double‐layer agarose method (Figure S2, Supporting Information). Lox enzymes positioned at the C terminus of Soc were displayed on the surface of T4 via affinity binding of Soc to phage capsid (T4‐Lox, TL). In comparison to the T4 phage, TL demonstrated a slight increase in dimensions, measuring a length of 240 nm and a width of 112 nm (Figure S3, Supporting Information). Both T4 and TL exhibited surfaces with significant negative charges, as shown in Figure 1c. To achieve plasmid loading, TL was modified with the cationic polymer poly‐L‐Lysine. The zeta potential of poly‐L‐Lysine‐functionalized TL (TLL) transitioned from negative (−27.8 mV) to positive (32.0 mV) and the charge stability of prepared TLL was related to the incubation time (Figure S4, Supporting Information). Following plasmid adsorption, the zeta potential reverted to −27.4 mV (Figure 1c), signifying the successful preparation of T4‐Lox‐DNA (TLD). Ultimately, to prevent plasmid degradation, minimize the immunogenicity, and provide iron for the Fenton reaction, TLD was functionalized with dextran iron oral fluid (a drug for iron deficiency anemia) to form a T4‐Lox‐DNA‐Fe probe (TLDF). The results revealed that the assembly process of dextran iron was time‐dependent (Figure S5, Supporting Information). A layer of dextran iron with a thickness of 20 nm was formed on the TLD after assembly for 2 h (Figure 1d,e). In comparison to other iron‐containing nanoparticles, the preparation process of TLDF required only a brief stirring, which would facilitate the retention of Lox proteins. In addition, the hydrodynamic diameters of probes were consistent with the results of TEM (Figure 1f). The in vitro *s*tability experiment of TLDF was performed. It exhibited that the hydrodynamic diameters at 4 °C remained stable over a week (Figure S6, Supporting Information).

UV–vis spectra demonstrated that the characteristic absorption peaks remained unchanged during the probe preparation (Figure S7, Supporting Information). The high‐angle annular dark field (HAADF)‐mapping images and energy‐dispersive X‐ray spectroscopy (EDS) disclosed that the TLDF primarily consisted of N, Fe, P, O, and C (Figure 1g,h). Among them, the P element could be attributed to the exogenous plasmid, and the Fe element was mainly distributed around the phage. Simultaneously, the chemical composition and chemical states of TLDF were measured by X‐ray photoelectron spectroscopy (XPS). As shown in Figure 1i, the TLDF was mainly composed of C, N, O, P, and Fe elements. The high‐resolution spectra of iron showed that the binding energies of Fe^2+^ 2p_3/2_ and Fe^2+^ 2p_1/2_ were 709.14 and 722.18 eV, respectively, while the binding energies of Fe^3+^ 2p_3/2_ and Fe^3+^ 2p_1/2_ were located at 710.88 and 724.28 eV, respectively (Figure 1j). The valence state of iron ions in the TLDF consisted of divalent and trivalent in ratios of 26.03% and 73.97%, respectively (Table S1, Supporting Information). The loading content of iron on the TLDF was quantitatively determined using atomic absorption spectrophotometry and found to be 803 µg L^−1^ (5 × 10^11^ pfu, Figure S8, Supporting Information).

### 2.2 Construction and Expression of Soc–Lox Fusion Protein

**Figure 2:**
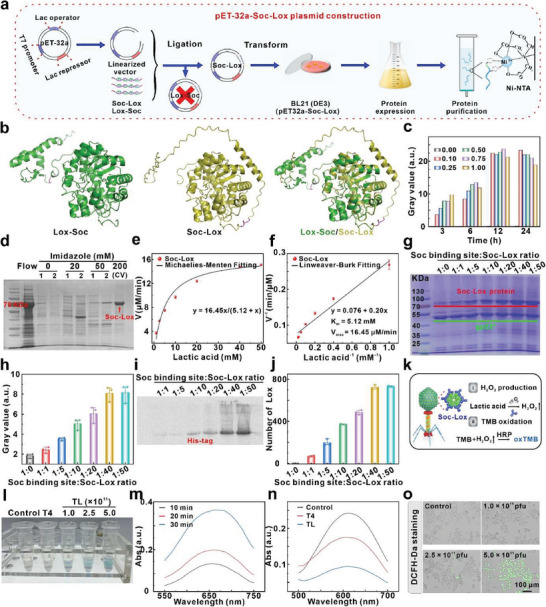
Construction and expression of Soc–Lox fusion protein. a) Schematic diagram of Soc–Lox plasmid construction and protein expression. b) The Lox–Soc (green color) and Soc–Lox (yellow color) fusion protein 3D structure model. c) Grayscale values of Soc–Lox protein expression induction by different IPTG concentrations. d) Soc–Lox protein purification. e) Michaelies–Menten and f) Lineweaver–Burk plot fitting of H_2_O_2_ production by the Soc–Lox fusion protein (*n* = 3). g) SDS‐PAGE analysis Soc–Lox protein display onto phage and h) grayscale values (*n* = 3). i) WB examination of the Soc–Lox fusion protein displayed on phage. j) Quantification of the number of Soc–Lox fusion proteins on each phage (*n* = 3). k), l) Schematic of TMB oxidation by H_2_O_2_ production by different amounts of TL and pictures of oxTMB solution (45 min). m) UV–vis absorption spectra of oxTMB and n) oxidation 2,6‐dichloroindophenol. o) DCFH‐DA fluorescence signals in the cells after treatment with different concentrations of TL.

Lactic acid assumes a pivotal role in the processes of tumor proliferation, metastasis, and recurrence. In response to the heightened demand for lactic acid in tumor tissues, we advocate for the construction of a lactate oxidase (Lox) delivery vehicle to disrupt the energy source of tumor cells. Both the N and C terminus of Soc are well exposed, allowing Soc suitable as an adapter to display foreign proteins on the T4 capsid. The *Lox* nucleotide sequence (GenBank: D50611.1) derived from *Aerococcus viridans* was inserted into the upstream or downstream sequence of the *Soc* gene to construct the Lox–Soc or Soc–Lox open reading frame (ORF).^[^ ^19^ ^]^ Surprisingly, the fusion protein exhibited a lack of catalytic activity when the Lox coding sequence was positioned at the N‐terminal of Soc (Lox–Soc, **Figure** **2**a). In contrast, Soc–Lox displayed high catalytic activity. The tFold program was employed to construct a 3D structure model of the fusion protein sequence from scratch (Figure 2b). Insights from 3D modeling revealed that a noticeable difference between the Lox–Soc and Soc–Lox fusion proteins lay in their exhibited reverse symmetry, and the Lox–Soc fusion protein had 1–2 smaller alpha helices in the Soc protein region, whose steric hindrance would affect the flexibility. The Soc–Lox nucleotide sequence was depicted in Figure S9a (Supporting Information) and validated through DNA sequencing using a T7 terminator primer (Table S2, and Figure S9b, Supporting Information). After that, *Soc–Lox* was ligated into the pET‐32a vector and expressed in the *E. coli* BL21 (DE3) strain.

Isopropyl β‐D‐thiogalactoside (IPTG), functioning as a lactose analog, was employed to regulate the expression of Soc–Lox protein. Various concentrations of IPTG (0–1 mm) and induction time (3, 6, 12, and 24 h) were performed (Figure S10a, Supporting Information). A positive correlation was observed between protein expression and IPTG concentration during the brief induction period (Figure S10b,c, Supporting Information). Nevertheless, beyond 12 h, protein expression exhibited a nearly independent relationship with IPTG concentration (Figure S10d, Supporting Information), and lower concentration (0.1 mm) was determined to be effective in attaining elevated levels of protein expression (Figure 2c; Figure S10e, Supporting Information). The Soc–Lox fusion protein with a His‐tag was purified using the Ni‐NTA resin affinity chromatography method.^[^ ^20^ ^]^ Miscellaneous proteins were progressively eliminated by adjusting the imidazole concentration, where a purer fusion protein band was observed at 70 kDa when the imidazole concentration reached 200 mm (Figure 2d). Employing a His‐tag antibody for western blot (WB) analysis unveiled a distinct signal indicative of antibody binding on the PVDF membrane (Figure S11, Supporting Information). Additionally, mass spectrometry of the purified protein identified a signal peak at 66.4 kDa (Figure S12, Supporting Information). These results confirmed that the Soc–Lox protein has been successfully constructed, expressed, and purified.

In assessing the catalytic efficacy of the Soc–Lox fusion protein, we determined the steady‐state kinetic parameters for Soc–Lox. Various concentrations of lactic acid (1.0, 2.5, 5.0, 10, 20, 40, and 50 mm) were employed as substrates. We measured the production rate of H_2_O_2_ during the reaction, and the substrate concentration, along with the corresponding reaction rate, was fitted using the Michaelis–Menten equation (Equation 1, Figure 2e). Additionally, Lineweaver‐Burk fitting (Equation 2) was carried out by plotting the reciprocal of the substrate concentration against the reciprocal of the reaction rate (Figure 2f). The results indicated that the Soc–Lox fusion protein exhibited a Michaelis constant (*K*_m_) of 5.12 mm and a maximum reaction velocity (*v*_max_) of 16.45 µM min^−1^.

(1) $$\;v=v_{max}*\left[s\right]/\left(k_{m}+\left[s\right]\right)$$

(2)

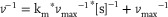




Soc, functioning as an aptamer for in vitro enzyme display, presents more substantial advantages when compared to traditional loading technologies. This includes a greater loading capacity and increased flexibility in modulation potentials. To identify the optimal assembly molar ratio of Soc–Lox for the enzyme display, we calculated the molarity of the Soc site according to Avogadro's constant (Equation 3). Soc–Lox fusion proteins, featuring diverse molar ratios (ranging from 1:0 to 1:50), were introduced to the enzyme display, and a gradual rise in the display of Soc–Lox protein on the phage as the molar ratio increased (Figure 2g). Notably, the maximum number of displayed proteins was attained at a molar ratio of 1:40, as depicted in Figure 2h. The displayed Soc–Lox was specifically detected by WB, revealing that the antibody signal progressively increased with an elevated molar ratio, reaching a substantial binding signal at a molar ratio of 1:40 (Figure 2i). These experimental findings substantiated the successful display of Soc–Lox. T4 as a vehicle can accurately quantify the number of enzyme molecules displayed on each phage based on the number of gp23* (930 copies) (Equation 4). The quantification of Soc–Lox indicated that 720 Lox molecules were displayed at a 1:40 molar ratio (Figure 2j), which was below the maximum assembly capacity of 870. It was also observed that the quantity of Soc–Lox fusion proteins on phage remained relatively constant even with an increased molar ratio of 1:50 (729). This phenomenon could be attributed to spatial site resistance, which restricted the number of Soc–Lox bindings. In conclusion, the developed phage delivery vehicle enables precise control of the protein number by adjusting the feeding ratio within a specified range. The Soc aptamer exhibited high efficiency, loading enzymes within a mere 30 min without the need for additional reagent assistance.

(3) $$\mathrm{Mol}e_{\left(\mathrm{Soc}\right)}=\left(N^{*}870\right)/N_{A}$$

where N was the number of T4, 870 was the number of Soc sites, and N_A_ was Avogadro's constant.

(4) $$\mathrm{Numbe}r_{\left(\mathrm{Soc}-\mathrm{Lox}\right)}={\left(\mathrm{Grayscal}e_{\left(\mathrm{Soc}-\mathrm{Lox}\right)}/\mathrm{Grayscal}e_{\left(\mathrm{gp}23^{*}\right)}\right)}^{*}930$$

where Grayscale_(Soc–Lox)_ and Grayscale_(gp23*)_ were the grayscale of Soc–Lox and the major capsid protein gp23* corresponding to the SDS‐PAGE result, respectively, and 930 was the number of gp23*.

The Soc–Lox displayed on the phage was designed to deplete lactic acid and produce H_2_O_2_. The catalytic properties were assessed (Figure 2k; Figure S13a, Soc‐Lox), and the content of H_2_O_2_ exhibited a remarkable increase with the TL number (Figure 2l). In the absence of Lox, however, no H_2_O_2_ was detected (Figure S13b, Supporting Information). The increase in the absorbance of oxTMB at 660 nm indicated that TL enzymatic activity produced H_2_O_2_ in a time‐dependent manner (Figure 2m). To monitor the redox interaction of TL with lactic acid, phenoxazine methyl sulfate (PMS, a yellow solution) was employed as an electron transfer agent. This agent is capable of receiving transferred electrons within the enzymatic reaction and transferring them to 2,6‐dichloroindophenol (DCIP, a blue solution). The color of DCIP gradually faded and completely disappeared after 5 min (Figure S14), and the absorbance of DCIP at 600 nm steadily decreased (Figure 2n). 2,7‐dichlorodihydrofluorescein diacetate (DCFH‐DA) was employed as an indicator to track the catalytic activity of TL in cells. As shown in Figures 2o and S15 (Supporting Information), the green fluorescence signal in 4T1 cells was increased with the TL, indicating the enzyme vehicle retained its catalytic potential in the complicated intracellular environment.

### 2.3 Construction of a Recombinant DNA Regulatory System with an Oxygen‐Sensing Function

**Figure 3:**
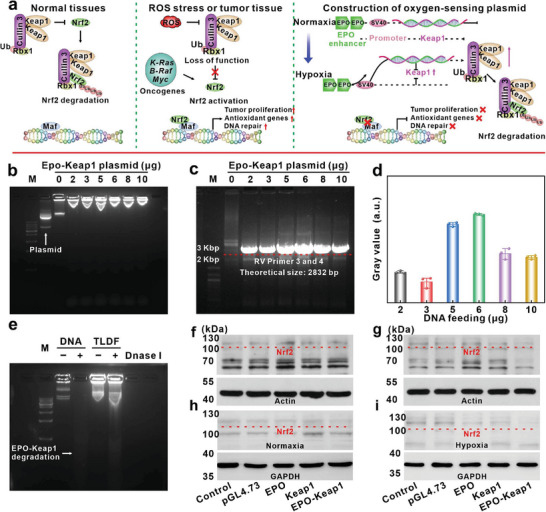
Construction of a recombinant DNA regulatory system with an oxygen‐sensing function. a) The working principle of oxygen‐sensing EPO‐Keap1 plasmid. b) Agarose gel results of probe loading EPO‐Keap1 plasmids. c) RV primer 3 and RV primer 4 PCR amplification bands and d) corresponding grayscale values (*n* = 3). e) The result of naked EPO‐Keap1 plasmid and TLDF probe digestion by *DNase I* enzyme. WB analysis of Nrf2 protein degradation by different plasmids at 24 h f) or 48 h g) in an anoxic environment. Under normoxia h) or hypoxia i) conditions degradation of intracellular Nrf2 by different plasmids.

While tumor cells exhibit active metabolic activity with numerous sources of ROS, Nrf2 stands out as a key transcription factor responsible for intracellular antioxidant activity.^[^ ^21^ ^]^ Nrf2 regulates the expression of antioxidant genes, thereby shielding tumors from ROS attacks in tumor tissues. In normal physiological conditions, Keap1 is responsible for tightly regulating Nrf2 activity, whereas the prevalence of Nrf2 gain or Keap1 loss is observed in tumor tissues (**Figure** **3**a). Hypoxia will enhance EPO gene transcription levels under physiological conditions.^[^ ^22^ ^]^ Inspired by this, a recombinant plasmid with an oxygen‐sensing function was constructed. This construction involved the insertion of a tandem repeat EPO enhancer upstream of the SV40 promoter of the pGL4.73 plasmid (Figure 3a). Under normal tissues, the EPO enhancer remained silent and did not activate downstream SV40 promoter transcription.

However, the EPO enhancer will activate in the hypoxic TME, resulting in the upregulation of downstream Keap1 gene expression (GenBank: AB020063.1) and downregulation of the Nrf2 signaling pathway through the Keap1‐Nrf2 axis.

We investigated the optimal assembly conditions of EPO‐Keap1 onto the surface of TL (5 × 10^11^ pfu) with varying plasmid contents (2, 3, 5, 6, 8, and 10 µg). Agarose gel electrophoresis results indicated a gradual increase in fluorescence signal with increasing plasmid feeding, reaching a maximum at 6 µg (Figure 3b). The prepared TLD probe served as the template and PCR amplification was conducted with reporter vectors primer 3 (RV primer 3) and primer 4 (RV primer 4) of pGL4.73 plasmid. The position of amplification bands was located between 2 and 3 Kbp, which was consistent with the anticipated size of 2832 bp (Figure 3c), indicating that the EPO‐Keap1 plasmid had been successfully loaded on the poly‐L‐lysine modified TL. Quantitative analysis revealed the highest amount of amplified nucleic acid sequence at a feeding concentration of 6 µg (Figure 3d). Additionally, Keap1 protein‐specific primers were also designed (Table S2, Supporting Information), and agarose gel electrophoresis further supported the aforementioned results (Figure S16, Supporting Information). During gene therapy, exposed DNA molecules were susceptible to rapid degradation by nucleases in the serum, leading to the loss of their function. To address this issue, the TLD was modified by dextran iron (TLDF). In vitro nuclease degradation experiments using DNase I demonstrated that the naked DNA was completely degraded, while the plasmid in TLDF remained intact (Figure 3e).

To validate the inhibition of the Nrf2 signaling pathway by the EPO‐Keap1 plasmid in cells, several plasmids were designed, including pGL4.73, pGL4.73‐EPO, pGL4.73‐Keap1, and pGL4.73‐EPO‐Keap1. WB results under hypoxic conditions for 24 h showed no significant change in the glycosylation‐modified Nrf2 (110 kDa) with different plasmid regulation (Figure 3f), indicating that the plasmid had no immediate effect (**F**igure S17, Supporting Information), while, with the treatment time extended to 48 h, significant degradation of Nrf2 proteins was observed in the EPO‐Keap1 group (Figure 3g). The oxygen‐sensing capability of the plasmids was also investigated. Under normal oxygen concentrations, the EPO enhancer remained inactive, and the EPO‐Keap1 plasmid exhibited no activity (Figure 3h). Conversely, in a hypoxic environment, the EPO enhancer promoted Keap1 expression and led to significant degradation of the Nrf2 protein (Figure 3i; Figure S18, Supporting Information).

### 2.4 TLDF Cascade Reaction Generation Hydroxyl Radicals

**Figure 4:**
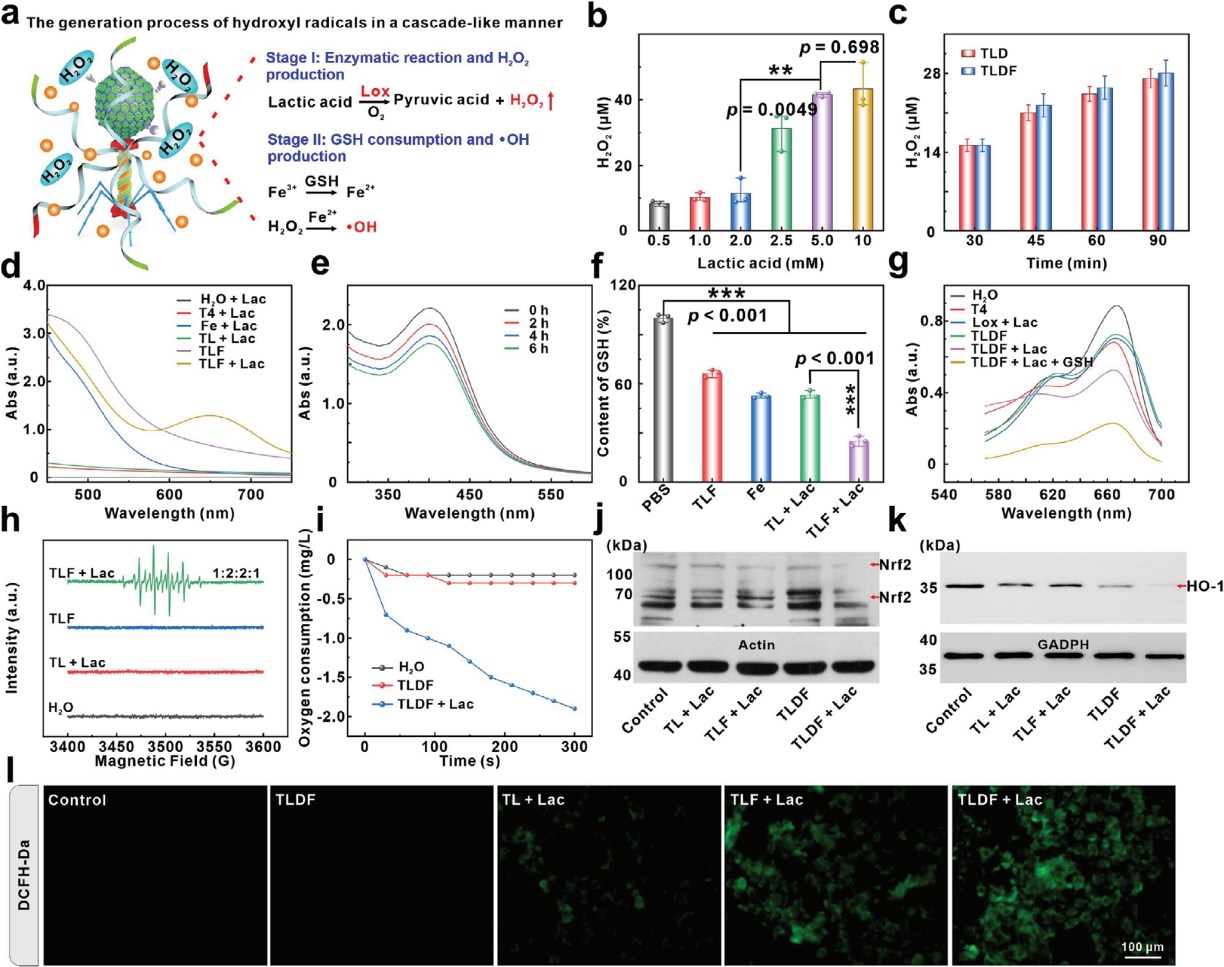
TLDF probe cascade reaction generation hydroxyl radicals. a) The generation process of •OH in a cascade‐like manner. b) H_2_O_2_ production by the TLDF with different lactic acid concentrations (*n* = 3). c) The amount of H_2_O_2_ production by the TLD and TLDF with lactic acid at 5 mm (*n* = 3). d) UV–vis absorption spectra of oxTMB. e) The amount of GSH remaining in the solution after GSH incubation with TLDF for different times and f) total GSH content in 4T1 cells after incubation with different probes (*n* = 3). g) MB detection of •OH production by different probes. h) Electron spin resonance spectra of •OH trapped by the DMPO. i) Changes in dissolved oxygen concentration in solution during Lox enzymatic reactions. WB detection j) Nrf2 and k) HO‐1 protein content after different treatments. l) Fluorescence images of DCFH‐DA‐stained 4T1 cells incubated with different probes.

As a catalyst, dextran iron conferred phage vehicle with a Fenton reaction ability. Hydroxyl radicals were generated by the TLDF in a cascade‐like manner (**Figure** **4**a). To determine the optimal substrate concentration, TLDF was incubated with various concentrations of lactic acid (0.5, 1.0, 2.0, 2.5, 5.0, and 10 mm). An increase in lactic acid concentration led to a gradual rise in H_2_O_2_ production, reaching a plateau of 41 µm at 5 mm (Figure 4b). Metal ions are known to bind to the enzyme's active site, potentially inhibiting enzyme activity. There was a minimal disparity in H_2_O_2_ production at equivalent substrate concentrations, suggesting dextran iron has negligible influence on Lox (Figure 4c). A distinct oxidized TMB (oxTMB) absorption peak emerged through a cascade‐like reaction (Figure 4d). Fe^3+^ ions were reduced to Fe^2+^ by GSH, and the remaining GSH gradually declined, resulting in a decrease in UV–vis absorption at 412 nm (Figure 4e). In addition, total glutathione (GSH) levels in cells were also investigated. Intriguingly, the TL + Lac group demonstrated the ability to deplete intracellular GSH even in the absence of Fe elements (Figure 4f). Conceivably, a fraction of the GSH could have been consumed by the H_2_O_2_ produced by the TL, and the TLF + Lac group exhibited a significant depletion of GSH, which could be associated with the valence change of Fe^3+^/Fe^2+^ (Figure 4f). The ability of the TLDF to deplete GSH and enhance •OH production was additionally investigated using methylene blue (MB). The absorption peak at 650 nm in the TLDF + Lac group was markedly diminished in the presence of GSH (Figure 4g). Subsequently, 5,5‐dimethyl‐1‐pyrroline‐N‐oxide (DMPO) served as the capture reagent to identify the type of ROS through electron spin resonance spectroscopy (ESR). As shown in Figure 4h, a 1:2:2:1 DMPO‐OH signal peak was seen in the TLF + Lac group, indicating that H_2_O_2_ produced by Lox could potentially generate •OH with dextran iron.

The enzymatic reaction can effectively interact with the EPO‐Keap1 plasmid. Throughout the enzymatic reaction, there was an observed decline in dissolved oxygen by 1.9 mg L^−1^ (Figure 4i), fostering a positive feedback regulation of EPO‐Keap1 expression. The degradation of Nrf2 was found in the TLDF + Lac group compared with the TLDF group (Figure 4j; Figure S19, Supporting Information). To verify this hypothesis, tris(4,7‐biphenyl‐1,10‐o‐phenanthroline) ruthenium dichloride served as an oxygen‐sensitive dye to detect intracellular oxygen concentration. The high concentration of oxygen quenched the fluorescence in the TLDF group, whereas a prominent fluorescent signal was observed in the TLDF + Lac group (Figure S20, Supporting Information). Nrf2 regulates the expression of heme oxygenase‐1 (HO‐1), providing resistance to “ferroptosis” in tumor cells. The notable down‐regulation of intracellular HO‐1 was attributed to a reduction in Nrf2 in the TLDF + Lac group (Figure 4k; Figure S21, Supporting Information). The synergistic “two‐pronged” strategy involving the Fenton reaction (an increase of free radicals) and EPO‐Keap1 (inhibition of free radical scavenging) in TLDF was assessed using DCFH‐DA. A more intense fluorescent signal was observed in the TLDF + Lac group, indicating that the EPO‐Keap1 plasmid suppressed the expression of antioxidant genes, leading to the accumulation of •OH in the cells (Figure 4l).

### 2.5 TLDF Phagocytosis and Therapy In Vitro

**Figure 5:**
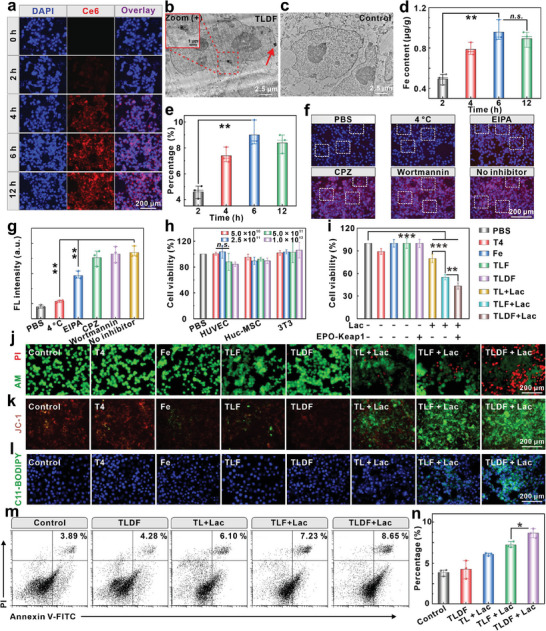
TLDF phagocytosis and therapy in vitro. a) Fluorescence imaging of Ce6 after 4T1 cells incubation with TLDF@C probe at different times. b) Ultrathin sections of 4T1 cells after incubation with or c) without the TLDF. d) Atomic absorption spectroscopy detection intracellular Fe content and e) percentage of phagocytosis after 4T1 cells incubation with the TLDF at different times (*n* = 3). Fluorescence imaging f) and intracellular mean fluorescence intensity g) of TLDF@C probes phagocytosed by 4T1 cells treated with different cellular pathways inhibitors (CPZ: chlorpromazine) (*n* = 3). h) Cell viability of HUVEC, Huc‐MSC, and 3T3 cells after incubation with the TLDF (*n* = 5). i) Cell viability of 4T1 after different treatments (*n* = 4). j) Fluorescence imaging of Calcein‐AM/PI‐stained 4T1 cells after different treatments. Fluorescence imaging of k) JC‐1 and l) C11‐BODIPY after 4T1 cells treated by different probes. m,n) Flow cytometry detection apoptosis cells after different treatments and percent of apoptosis cells (*n* = 3).

Chlorin e6 (Ce6)‐labeled TLDF (TLDF@Ce6) was incubated with 4T1 cells for different times (0, 2, 4, 6, and 12 h) to investigate the mechanism of probe entry into cells. The probe gradually permeated the cell membrane, reaching its peak fluorescence intensity after 6 h (**Figure** **5**a). The TLDF was observed in the 4T1 cells through the ultrathin TEM section (Figure 5b), whereas no probe was observed in the control group (Figure 5c). Atomic absorption spectroscopy was used to detect the intracellular iron content and phagocytosis efficiency, and the results showed that the iron ions peaked at 957.85 ng g^−1^ (Figure 5d), with phagocytosis efficiencies of 4.60%, 7.39%, 9.00%, and 8.39% at 2, 4, 6, and 12 h, respectively (Figure 5e). The optimal incubation period was determined to be 6 h. We further investigated the mechanism of endocytosed probes using various cellular pathway inhibitors, including chlorpromazine hydrochloride (CPZ, clathrin inhibitor), wortmannin (PI3K‐AKT inhibitor), EIPA (Na^+^‐H^+^ exchange channel and micropinocytosis inhibitor), and treatment at 4 °C (Figure 5f). As shown in Figure 5g, low temperature affected the energy‐mediated phagocytosis process. Meanwhile, the blockage of Na^+^‐H^+^ ion exchange channels and macropinocytosis (EIPA) greatly reduced intracellular fluorescence. Therefore, TLDF nanocarriers transported enzymes and plasmids into cells mainly through micropinocytosis‐mediated endocytosis, and the transmembrane process required energy participation.

In the course of transportation, the probes inevitably engage with the circulatory system. Human umbilical vein endothelial cells (HUVEC), human umbilical cord mesenchymal stem cells (HUC‐MSCs), and 3T3 cells (mouse fibroblast) were utilized as model cells to investigate the cytotoxicity of the probes. All cells demonstrated high viability at elevated concentrations of TLDF (5 × 10^11^ pfu mL^−1^), indicating its minimal harm to the vascular system or fibroblast (Figure 5h). The anti‐tumor efficacy of TLDF on 4T1 cells was assessed. Lactic acid played a pivotal role in initiating TLDF, unlocking the “Pandora's Box” of H_2_O_2_ generated by Lox, subsequently converted to •OH through a cascade‐like process. However, Nrf2 upregulated antioxidant gene expression (NQO1, GCLC, and SLC7A11), which counteracted the •OH detrimental effects (Figure 5i). The antioxidant braking strategy augmented the cytotoxic effect, resulting in a robust PI signal in the nucleus (Figure 5j). The status of mitochondrial membrane potential (∆ψ_m_) was detected by the JC‐1. The TL, TLF, and TLDF groups induced depolarization of the mitochondrial membrane potential in the presence of lactic acid (green fluorescence signal). The downregulation of Nrf2 in the TLDF group significantly heightened membrane depolarization, resulting in mitochondrial dysfunction (Figure 5k).

Ferroptosis, a form of iron‐dependent cell death driven by lipid peroxidation, was monitored by the C11‐BODIPY. As shown in Figure 5l, the Lox enzymatic reaction did not elicit an indicative of ferroptosis. However, dextran iron made the phage vehicle with efficient Fenton catalytic ability, and the tumor cell membrane was subjected to lipid peroxidation (TLF + Lac). The enzymatic reaction, effectively interacting with the EPO‐Keap1 plasmid, disrupted redox homeostasis, and C11‐BODIPY‐labeled fluorescence was more pronounced upon stimulation with TLDF + Lac (Figure 5l). To demonstrate ferroptosis‐induced tumor cell death of the TLDF, different ferroptosis inhibitors, including deferoxamine mesylate (DFO, an iron chelating agent), GSH, and *N*‐acetyl‐L‐cysteine (NAC, a precursor of GSH biosynthesis) were added to investigate the effect on cell viability. The results showed that the DFO significantly inhibited the therapeutic effect of TLDF by chelating with iron ions released from the TLDF (Figure S22, Supporting Information). GSH overexpression in tumor tissue maintains growth‐required redox homeostasis, and cell viability was enhanced when GSH was supplemented compared with without inhibitor group. NAC also exhibited the cytoprotective effect by indirectly increasing GSH content. The fluorescence signals of Annexin‐V and PI were analyzed using flow cytometry (Figure S23, Supporting Information). In a two‐pronged synergistic treatment strategy, the percentage of necrotic cells increased from 7.23% to 8.65% (Figure 5m). Importantly, the EPO‐Keap1 plasmid remained inactive in the absence of lactic acid, resulting in no significant difference in apoptosis between the TLF and TLDF groups (Figure S24, Supporting Information). The primary factor contributing to the functionality of the designed EPO enhancer was its sensitivity to oxygen concentration, enabling its operation only in a hypoxic environment (Figure 5n).

### 2.6 Transcriptome Analysis of the Mechanism of TLDF‐Mediated Ferroptosis in Cells

**Figure 6:**
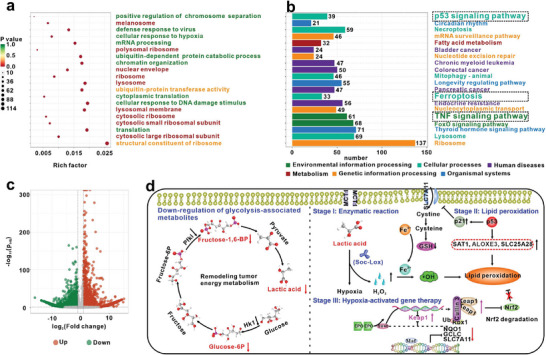
Transcriptome analysis of 4T1 cells after treated by TLDF. a) Top 20 enrichment in GO term enrichment analysis and b) KEGG pathway enrichment analysis of differentially expression genes after 4T1 cells treatment with TLDF probe. c) Volcano plotting of transcript expression in 4T1 cells induced by the TLDF. d) Schematic diagram of TLDF‐induced changes in intracellular metabolism.

Transcriptomic analysis was conducted to achieve a comprehensive comprehension of cellular pathways and potential molecular mechanisms. Gene Ontology (GO) was employed to analyze the biological processes, cellular components, and molecular functions of the differentially expressed genes. Differential genes were found to be implicated in diverse biological processes, such as cell apoptosis, mitochondrial function, and DNA binding. The expression levels of 38 and 95 genes, respectively, enriched in the cellular response to hypoxia and DNA damage stimuli, were significantly altered (**Figure** **6**a). KEGG enrichment analysis revealed that 33 differentially expressed genes were enriched as regulators of the ferroptosis pathway (Figure 6b). Furthermore, 39 genes were identified to be involved in the p53 signaling pathway, and 61 genes participated in the TNF signaling pathway. This discovery supports previous research highlighting the significance of the p53 and TNF signaling pathways in ferroptosis.^[^ ^23^ ^]^ Examination of the top 20 signaling pathways unveiled that around 12.86% of those genes were associated with the regulation of ferroptosis.

The RNA sequencing results showed that 9259 differentially expressed genes were identified in 4T1 cells stimulated by the TLDF + Lac compared with the control groups (*P_adj_* < 0.05 and |log_2_ fold change| > 1). Among them, 4136 genes exhibited up‐regulation, while 5123 genes underwent downregulation (Figure 6c). Tumor cells predominantly rely on glycolysis for energy, and there is a significant increase in the synthesis of the essential molecule lactic acid in various solid tumors.^[^ ^24^ ^]^ Previously reported results have shown that Lox can effectively deplete lactic acid and block the energy source of tumor cells. In addition, lactate depletion strategies can alter energy metabolism to enhance tumor therapeutic efficacy. Transcriptome analysis demonstrated that glycolysis‐related enzymes, such as hexokinase (HK1) and phosphofructokinase (PFKl, *p* = 1.37 × 10^−8^), responsible for catalyzing the production of fructose 1,6‐diphosphate, were markedly downregulated (Figure 6d). Concurrently, isocitrate dehydrogenase (IDH3α), an enzyme integral to the tricarboxylic acid cycle, and succinyl‐CoA synthetase (SUCLA2), involved in the production of citric acid, exhibited upregulation (Figure 6d). According to these findings, it appeared that the lactate depletion strategy has altered the metabolic profile of tumor cells. This could be attributed to Lox exerting an inhibitory effect on lactic acid energy metabolism, compelling tumor cells to derive energy through the TCA cycle.

Transcriptome analysis unveiled both positive and negative regulation of ferroptosis by the p53 signaling pathway. The upregulated expression of genes, such as spermidine/spermine N1‐acetyltransferase 1 (SAT1), arachidonic acid lipoxygenase 3 (ALOXE3), and SLC25A28, has been associated with the promotion of lipid peroxidation (Figure 6d). Conversely, p53 has been observed to suppress the expression of System XC‐ (SLC7A11) by upregulating the downstream CDKN1A gene (p21, *p* = 7.81 × 10^−7^). In the Lox enzymatic reaction, the EPO enhancer detected low oxygen levels and subsequently increased Keap1 expression. The transcriptome analysis revealed a significant downregulation in the expression of Nrf2‐regulated antioxidant genes, such as NAD(P)H: quinone oxidoreductase 1 (NQO1), glutamate–cysteine ligase catalytic subunit (GCLC), and SLC7A11 (Figure 6d). In conclusion, the EPO‐Keap1 signaling pathway produced an “antioxidant braking effect”, amplifying the oxidative environment, while TLDF could also modify the metabolic pattern of tumor cells, inducing ferroptosis.

### 2.7 TLDF Safety Evaluation In Vivo

Diverse factors, including the immunogenicity and physicochemical properties of probes, can lead to hemolysis. The hemolytic properties of T4, TL, and TLDF were evaluated before in vivo testing. The hemoglobin was spilled out from the cells in water, serving as a positive control group (Figure S25,Supporting Information inset). In contrast, prepared T4, TL, and TLDF probes showed essentially identical absorbance at 560 nm to the PBS group (Figure S25a, Supporting Information), indicating that they did not induce hemolysis. The morphology of erythrocytes remained unaltered, maintaining their distinctive two‐sided concave structure (Figure S25b, Supporting Information). This result indicated that TLDF probes were safe for intravenous administration.

To evaluate both short‐term and cumulative toxicity of the TLDF, healthy mice were randomly divided into three groups: PBS, a single injection of TLDF, and multiple injections of TLDF (every five days, for six injections in total). At the molecular level, alanine aminotransferase (ALT), aspartate aminotransferase (AST), creatinine (Crea), and urea nitrogen (Urea) were examined. Statistically, there was no significant difference in the ALT and AST levels between the experimental group and control group (Figure S26a, Supporting Information), both remained in the normal range. Additionally, Crea and Urea Crea and Urea were effectively filtered by the kidneys’ glomeruli and tubules (Figure S26a, Supporting Information). These findings revealed that the phage vehicle camouflaged with dextran iron was safe for the kidneys and liver.

White blood cells (WBC) are the main component of the immune system, crucial for the body's defense against infections and diseases. No evidence of an inflammatory reaction was observed in the WBC measurements following TLDF delivery, which remained within the normal range (Table S3, Supporting Information). There was a minimal change in the number of erythrocytes and platelets, and no significant release of hemoglobin was observed (Figure S26b, Supporting Information). In addition, other routine blood parameters, such as the number of lymphocytes, monocytes, neutrophils, erythrocyte cumulative pressure, and mean erythrocyte volume were within healthy ranges (Table S3, Supporting Information). The organ index was calculated for critical organs such as the heart, liver, lung, and kidney. The results indicated that the administration of TLDF did not result in any adverse effects on organ function (Figure S26c, Supporting Information). Hematoxylin‐eosin staining (H&E), including the heart, liver, kidney, spleen, lung, and small intestine was performed to assess the impact of TLDF on tissue structure and cell morphology. These organs exhibited normal morphology and well‐defined histology, without any signs of organ damage, degeneration, or necrosis (Figure S26d, Supporting Information).

### 2.8 Immunogenicity and Tumor Targeting of TLDF In Vivo

**Figure 7:**
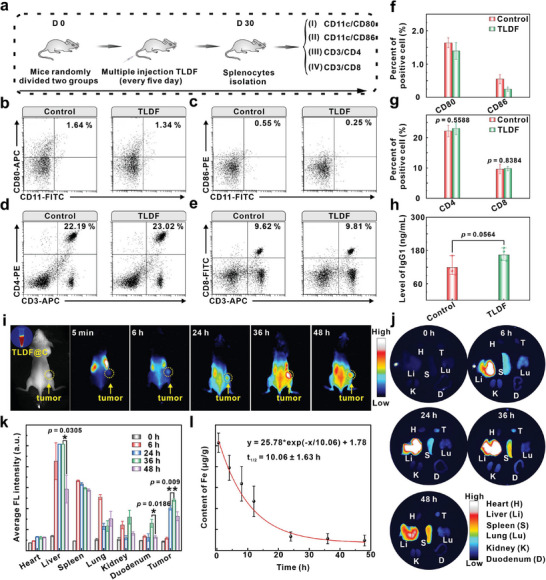
Immunogenicity and tumor targeting of TLDF in vivo. a) Schematic illustration showing the treatment steps and procedures for evaluating the immunogenicity of TLDF probe in Balb/C mice. b,c) Representative flow cytometry analysis of the population of DC maturation (CD11c/CD80, CD11c/CD86) in the spleen after treatment with TLDF on day 30. Representative flow cytometry analysis of the population maturation of d) CD4^+^ T cells (CD3/CD4) and e) CD8^+^ T cells (CD3/CD8) in the spleen after treatment with TLDF on day 30. f) Quantitative data of the population of DC maturation (*n* = 4). g) Quantitative data of the population of CD4^+^ T cells and CD8^+^ T cells (*n* = 4). h) ELISA detection IgG1 content in serum after mice treatment with TLDF on day 30 (*n* = 4). i) Representative fluorescence images of 4T1 tumor‐bearing mice at various time points post tail vein injection of TLDF@C. j) Representative fluorescence images of major organs at different time points and k) corresponding fluorescence intensities at various time points post tail vein injection of TLDF@C (*n* = 3). l) Pharmacokinetics of Fe during blood circulation in healthy mice after injection of the TLDF (*n* = 3).

The capacity of the TLDF nanocarrier to evade immune system recognition was crucial for tumor targeting. Healthy mice were randomly divided into two groups (*n* = 4) to explore the immunogenicity of TLDF. One group received the TLDF injections (**Figure** **7**a), while the other group was injected with PBS. Spleen samples were collected after immune stimulation, and the TLDF group showed no splenomegaly (Figure S27, Supporting Information). When exposed to exogenous antigens, antigen‐presenting cells, particularly dendritic cells (DCs), will upregulate the co‐stimulatory molecules CD80 and CD86. To explore the expression of co‐stimulatory factors in antigen‐presenting cells, a gating strategy was employed to eliminate interference from dead and adherent cells (Figure S28a, Supporting Information). The percentage of CD11/CD80 and CD11/CD86 in the TLDF group decreased from 1.64% to 1.34% (Figure 7b; Figure S28b, Supporting Information) and 0.55% to 0.25% (Figure 7c; Figure S28c, Supporting Information), respectively, compared to the PBS group. Helper T cells (CD3/CD4) and cytotoxic T cells (CD3/CD8) are also essential components of the immune response. Spleen cells were co‐stained with CD3, CD4, and CD8 antibodies to gain more insight into the T lymphocytes (Figure S29, Supporting Information). The CD3/CD4 and CD3/CD8 cell populations increased by 0.83% (Figure 7d) and 0.19% (Figure 7e), respectively, following TLDF treatment. However, there was no significant difference in the counts of antigen‐presenting cells (Figure 7f) and *T* lymphocytes (Figure 7g). Furthermore, IgG1 is a crucial immunoglobulin that plays a pivotal role in humoral immunity, contributing to pathogen neutralization and complement system activation. The levels of immunoglobulin IgG1 in the serum were detected by enzyme‐linked immunosorbent assay (ELISA). The results showed that the content of IgG1 did not significantly increase stimulated by the TLDF (Figure 7h). Dextran iron was an effective disguise material, making phage protein vehicles exhibit immune inertia.

The Cy5.5‐labeled TLDF (TLDF@C) was used for imaging to investigate the in vivo circulatory dynamics of the TLDF (Figure 7i, inset). Following a 6 h intravenous injection, the fluorescent signal gradually appeared at the tumor site and reached the peak fluorescent signal at 36 h (Figure 7i). To visualize the in vivo distribution of TLDF@C, major organs, including the heart, liver, spleen, lung, kidney, small intestine, and tumor were collected at different time points (0, 6, 24, 36, and 48 h). TLDF@C underwent a “first‐pass effect” attributed to the liver's vital detoxification role, resulting in notable accumulation in the liver (Figure 7j). The TLDF@C suffered a “first‐pass effect”, leading to significant accumulation in the liver. However, after 48 h, the signal in the liver gradually decreased (Figure 7k). The small intestine also contributed to probe metabolism and aimed the excretion of TLDF@C through excrement or urine (Figure S30, Supporting Information). The blood half‐life of TLDF was determined by the atomic absorption spectrometer and calculated to be *t*_1/2_ = 10.06 h (Figure 7l). The hemodynamic parameters obtained from this study provided valuable insight into the pharmacokinetic properties of the TLDF probe.

### 2.9 Two‐Pronged Synergistic Treatment Strategy of TLDF In Vivo

**Figure 8:**
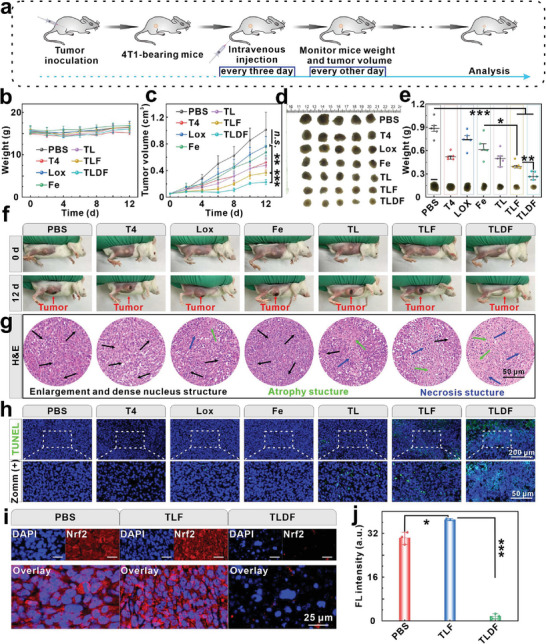
In vivo synergistic therapy of TLDF. a) Experimental outline showing the treatment procedures for evaluating the therapeutic outcomes in 4T1 tumor‐bearing mice. b) Body weight and c) tumor volume change after administering different probes (*n* = 5). d) Photographs of tumor tissue extracted from 4T1 tumor‐bearing mice after treatment with various probes and e) corresponding tumor tissue weight (Scale bar: 1 cm; *n* = 5). f) Photographs of 4T1 tumor‐bearing mice before (0 d) and after (12 d) treatment with various probes. Representative g) H&E staining, h) TUNEL staining, and i) Nrf2 immunofluorescence staining of tumor sections collected from the mice receiving different treatments on day 3 (Scale bar: 25 µm). j) Quantitative data of the Nrf2 fluorescence staining (*n* = 3).

A tumor therapy protocol was devised based on the metabolism of TLDF (**Figure** **8**a). Mice bearing 4T1 tumors were randomly divided into seven groups, including PBS, T4, Lox, Fe, TL, TLF, and TLDF. The mice were administered different probes through the tail vein every three days. The changes in body weight and tumor volume of the mice were monitored every other day, and the body weight exhibited a significant decrease in the first two days. However, the body gradually exceeded the pre‐treatment level (Figure 8b), probably due to the organism requiring a recovery period following the administration of the anesthetic drug. The lactic acid environment at the tumor site triggered the TL to generate H_2_O_2_, leading to a remarkable 52% enhancement in tumor suppression (Figure 8c), and the TLF group enhanced the tumor inhibition rate by 12% by converting H_2_O_2_ into highly toxic •OH. In a two‐pronged synergistic treatment strategy, the TLDF group negatively regulated the Nrf2, resulting in effective tumor growth inhibition with a tumor inhibition rate of up to 78% (Figure 8c). The size of the dissected tumors (Figure 8d) and weight (Figure 8e) in different groups were consistent with the tumor volume growth curve, and the TLDF therapy regimen exhibited a significant inhibitory effect on tumor growth (Figure 8f). H&E was routinely employed for the pathological examination of tissues. The results revealed nuclear enlargement and irregular and tightly packed morphology in the control groups, indicating rapid proliferation of tumor cells. In the TLDF group, however, the nuclei were wrinkled, signifying substantial necrosis in the tumor tissues (Figure 8g).

TdT‐mediated dUTP Nick‐End Labeling (TUNEL) staining was additionally conducted to assess the therapeutic impact on tumors. Negligible fluorescence‐labeled signals in the PBS, T4, Fe, and Lox groups indicated that these probes did not cause DNA damage to tumor cells (Figure 8h). Compared with the TL and TLF groups, a strong TUNEL signal was observed in the TLDF group, which was consistent with the results of H&E staining. The expression levels of hypoxia‐inducible factor (HIF‐1α) at the tumor site were examined by immunofluorescence staining. The phage vector, camouflaged with dextran iron, effectively evaded clearance, allowing the Lox enzymatic reaction at the tumor site to induce an increase in HIF‐1α levels (Figure S31, Supporting Information). To explore the influence of the Nrf2 signaling pathway on tumor therapy, we conducted immunofluorescence staining of tumor sites using the Nrf2 antibody. Prominent expression of the Nrf2 protein in whole tumor tissue, indicating a correlation with accelerated tumor growth in the PBS group (Figure 8i). In the TLF group, ferroptosis increased tumor cell lipid peroxidation and the intracellular Nrf2 antioxidant signaling pathway also activated, resulting in a significant enhancement of fluorescence signal, which reduced the therapeutic efficacy (Figure 8j). The TLDF exhibited a significant reduction in Nrf2 protein expression through the EPO‐Keap1 plasmid. This result further supported the notion that TLDF could compromise the efficacy of antioxidant defense mechanisms in malignant cells and heighten their vulnerability to therapeutic interventions.

## 3 Conclusion

In this study, we reported a novel TLDF bio‐intelligent nanoprobe using T4 phage display technology and oxygen‐sensing plasmid to enhance CDT. The TLDF nanoprobes exhibited “personalized tumor therapy” characteristics upon delivery. First, a high concentration of lactic acid environment as a “key” triggered the Lox enzymatic reaction, leading to CDT through a cascade‐like reaction. Second, the hypoxia TME acted as the other “key”, activating the bio‐intelligent EPO‐Keap1 plasmid to interfere with the Nrf2 signaling pathway. Moreover, during the starvation therapy accompanied by reduced oxygen concentration, positive feedback regulation of EPO‐Keap1 expression was achieved. Transcriptomic analysis revealed that the TLDF synergistic treatment reversed the energy metabolism of tumors. The two‐pronged strategy of H_2_O_2_‐supplying CDT and gene‐regulated antioxidant brakes disrupted redox homeostasis and enhanced the therapeutic effect. More importantly, the TLDF exhibited good biocompatibility and low immunogenicity at the molecular, cellular, and tissue organ levels. The TLDF probe was the first to integrate phage enzyme display technology with an adaptive regulatory plasmid. This strategy combined synthetic biology and nanotechnology to provide a new idea for anti‐tumor therapy.

## 4 Experimental Section

### Materials

All chemicals and reagents were provided by commercial sources. N‐hydroxysuccinimide (NHS), 1‐(3‐Dimethylaminopropyl)‐3‐ethylcarbodiimide hydrochloride (EDC), chlorpromazine hydrochloride, wortmannin, and EIPA were obtained from Shanghai Aladdin Biochemical Technology Co., Ltd. Chlorin e6 (Ce6) was purchased from Shanghai Macklin Biochemical Co., Ltd. Mitochondrial membrane potential assay kit (JC‐1) was obtained from Beyotime. Mouse IgG1 ELISA kit was purchased from MULTISCIENCES (LIANKE) BIOTECH, Co., Ltd. DNA marker (100‐3000 bp), M9 minimal salts, 50× TAE buffer, ElL‐ABTS chromogenic reagent kit, and peroxidase (Rz > 3.0) were purchased from Sangon Biotech (Shanghai) Co., Ltd. Transcriptome sequencing analysis (RNA‐seq) was performed by Benagen. PCR primer synthesis and nucleic acid sequencing were synthesized by Beijing Tsingke Biotech Co., Ltd.

### Amplification of T4 Phage with Soc Capsid Protein Mutation

The Soc mutated T4 phage was kindly given by Prof. Song Gao from Jiangsu Ocean University. 250 µL of overnight culture of *E. coli* BL21 and 2.5 × 10^6^ pfu T4 phage was mixed with 150 mL of Luria–Bertani medium (LB). The mixture was incubated at 37 °C and 100 rpm until the solution became clear. The unlysed *E. coli* BL21 was removed through centrifugation at 5 000 rpm for 30 min. The amplified phage was collected by centrifugation at 34 000 g for 1 h in an ultrahigh‐speed centrifuge (XE90, Beckman). To further remove impurities and cell debris from the supernatant, 0.22 µm filter membranes were utilized. The precipitate of T4 phage was subsequently re‐suspended in PBS, followed by the preparation of 15% and 50% sucrose solutions for further purification. The purified T4 was dialyzed overnight in Tris‐Mg buffer (10 mm Tris‐HCl, 1 mm MgCl_2_, and 100 mm NaCl).

### Construction of Soc–Lox Recombination Plasmid

The Lox nucleic acid sequence (derived from *Aerococcus viridans*) was optimized according to the codon preference of *Escherichia coli*, and the optimized nucleic acid sequence was synthesized at General Biosystems (Anhui, China) Co., Ltd. Soc. Lox was constructed as a fusion protein. To improve the solubility of the fusion proteins, the Soc–Lox and Lox–Soc nucleic acid sequences were amplified via forward primer: 5’‐GACGACGACGACAAGGGTGGCTATGTTAATATTAAGACC‐3’ and reverse primers: 5’‐GTCGACGGAGCTTTAATATTCATAACCATACGG‐3’ and ligated to pET‐32a vector by seamless cloning kit (Vazyme, Nanjing). The recombinant pET‐32a‐Soc‐Loc (Soc–Lox) and pET‐32a‐Lox–Soc (Lox–Soc) plasmids were transformed into *E. coli* BL21 (DE3) for expression.

### Soc–Lox Protein Display Process in Phage

T4 (5 × 10^10^ pfu) was mixed with Soc–Lox protein at varying molar ratios (1:0–1:50). After incubation at room temperature (RT) for 30 min, T4‐Lox (TL) was collected by centrifugation at 16 000 g for 40 min. The TL was re‐suspended in 50 µL PBS and detected by SDS‐PAGE. Image J software was used to analyze the grayscale values of both the Soc–Lox protein and the gp23* protein of the phage. The number of Soc–Lox proteins displayed on each phage was calculated based on the number of gp23* proteins.

### Construction of EPO‐Keap1 Plasmid

The Keap1 nucleic acid sequence was derived from *Mus musculus*. To improve expression in mammalian cells, the *Keap1* nucleic acid sequence was optimized by codon‐optimization and synthesized at General Biosystems (Anhui, China) Co., Ltd. *Keap1* nucleic acid sequence was ligated to pGL4.73 by *Hind III* and *Xba I* digestion sites. The oxygen‐sensing EPO enhancer was located at the N‐terminal of the *Keap1* sequence. The constructed pGL4.73‐EPO‐Keap1 plasmid was transformed into *E. coli* TOP10 stain for amplification.

### Preparation and Characterization of TLDF

T4 (5 × 10^11^ pfu) was incubated with Soc–Lox protein (molar ratio 1:40) at RT for 30 min. Prepared TL was collected through centrifugation at 16 000 g for 40 min. A 2 mL of poly‐L‐lysine (5 mg mL^−1^) solution was used to re‐suspend the TL. After incubation at RT for 2 h, the mixture was centrifuged at 16 000 g to obtain TLL. The TLL was re‐suspended in deionized water and mixed with EPO‐Keap1 (6 µg) at RT for 30 min. The TLD was mixed with iron dextran (final concentration of 0.5 mg mL^−1^), and the mixture was agitated at 28 °C for 2 h. Subsequently, the TLDF probes were washed twice with PBS and stored at 4 °C.

### Preparation of TLDF@Ce6 Probe

Ce6 (2 mg mL^−1^) was dissolved in dimethyl sulfoxide (DMSO). EDC (3.6 mg mL^−1^) and NHS (2.6 mg mL^−1^) were gradually added to the reaction solution and stirred at 28 °C for 6 h. The activated Ce6‐NHS was stored at −20 °C and without further purification. T4 (5 × 10^11^ pfu) and Ce6‐NHS (100 µg mL^−1^) were shaken for 6 h. The solution was subjected to centrifugation at 16 000 g for 40 min. The obtained T4‐Ce6 was used for the preparation of TLDF@Ce6 probes following the guidelines of TLDF.

### Detection of Mitochondrial Membrane Potential

The overnight culture of 4T1 cells was washed twice with PBS and incubated with the probes in a serum‐free medium for 6 h. The cells in a complete medium containing lactic acid (2.5 mm) were incubated for 18 h. Cells were washed twice with PBS and added 1 mL of complete culture medium and 1 mL of JC‐1 staining working solution, and then incubated at 37 °C for 20 min. Upon completion of the incubation, the cells were washed twice with JC‐1 staining buffer. Finally, fluorescence microscopy was utilized to observe the intracellular JC‐1 signal.

### The Blood Half‐Life of TLDF

To remove iron interference in red cells. Healthy Balb/c mice were injected with PBS as a control group, and blood was taken at the same time point as the experimental group. The weight of blood was recorded and digested by HNO_3_ (Guaranteed reagent, GR). The atomic absorption spectrophotometer was used to measure the iron content in each sample. The amount of iron in the blood at different time points in the experimental group was calculated as follows.

(5) $$\mathrm{Content}\;\mathrm{of}\;\mathrm{Fe}\;(\mu g/g)\;=\;\frac{Fe_{\left(E\right)}\;-\;Fe_{\left(C\right)}}{M_{\left(\mathrm{Blood}\right)}}$$

Fe_(E)_ and Fe_(C)_ represent the iron content of the experimental and control groups at the same time. M_(Blood)_ represents the weight of blood.

### The Synergistic Therapeutic Effect of TLDF In Vivo

Female Balb/c mice (five weeks) were subcutaneously injected with 4T1 cells (1 × 10^6^) in the right hand side. Upon reaching a predetermined tumor volume, the mice were randomly divided into seven groups: PBS, T4, Lox, Fe, TL, TLF, and TLDF. A probe (6.25 × 10^12^ pfu Kg^−1^) was administered via the tail vein every three days. Iron and Lox concentration per administration was 803 µg L^−1^ and 720 molecules/phage, respectively. After 72 h of treatment, one mouse from each group was selected for euthanasia, and tumor tissues were immersed in a 4% paraformaldehyde for H&E staining, TUNEL, and Nrf2 immunofluorescence staining. The mice's body weight and tumor volumes were documented every other day. Upon completion of the treatment, all mice were humanely euthanized, and the tumor tissues were extracted, photographed, and weighed. All animal experiments were approved by the Animal Experimental Ethics Committee of Huazhong University of Science and Technology (IACUC Number: S904).

### Statistical Analyses

All quantified data were expressed as mean ± standard deviation. Statistical comparisons between the two groups were performed by a two‐way Student *t*‐test and multiple comparisons by a one‐way ANOVA. ^*^ *p* < 0.05, ^**^ *p* < 0.01, ^***^ *p* < 0.001, and *n.s*. means no statistically significant difference.

## Conflict of Interest

The authors declare no conflict of interest.

## Supporting information

Supporting Information

## Data Availability

The data that support the findings of this study are available in the supplementary material of this article.
