# Supplementary material for: Engineering Phage Nanocarriers Integrated with Bio‐Intelligent Plasmids for Personalized and Tunable Enzyme Delivery to Enhance Chemodynamic Therapy
Source: Adv Sci (Weinh). 2024 Apr 6;11(24):2308349. doi: 10.1002/advs.202308349 (PMC11199971; doi:10.1002/advs.202308349)
Supplement: Supplementary file 1 — Supporting Information [file ADVS-11-2308349-s001.pdf]

ADVANCED SCIENCE
Open Access

Supporting Information

for *Adv. Sci.*, DOI 10.1002/advs.202308349

Engineering Phage Nanocarriers Integrated with Bio-Intelligent Plasmids for Personalized and Tunable Enzyme Delivery to Enhance Chemodynamic Therapy

Xiao-Lin Hou, Bin Zhang, Kai Cheng, Fang Zhang, Xiao-Ting Xie, Wei Chen, Lin-Fang Tan, Jin-Xuan Fan, Bo Liu* and Qiu-Ran Xu**

Supporting information

Engineering Phage Nanocarriers Integrated with Bio-Intelligent Plasmids for Personalized and Tunable Enzyme Delivery to Enhance Chemodynamic Therapy

Xiao-Lin Hou, Bin Zhang, Kai Cheng, Fang Zhang, Xiao-Ting Xie, Wei Chen, Lin-Fang Tan, Jin-Xuan Fan, Bo Liu* and Qiu-Ran Xu**

X.-L. Hou, B. Zhang, K. Cheng, F. Zhang, X.-T. Xie, W. Chen, L.-F. Tan, J.-X. Fan, B. Liu

Britton Chance Center for Biomedical Photonics at Wuhan National Laboratory for Optoelectronics - Hubei Bioinformatics & Molecular Imaging Key Laboratory, Department of Biomedical Engineering, College of Life Science and Technology, Huazhong University of Science and Technology, Wuhan 430074, Hubei, P. R. China.

E-mail address: chen1980wei@mail.hust.edu.cn (W. Chen), lbyang@mail.hust.edu.cn (B. Liu), Fax: (+) 86 27-8779-2202

B. Liu

Key Laboratory of Biomedical Photonics (HUST), Ministry of Education, Huazhong University of Science and Technology, Wuhan 430074, Hubei, P. R. China.

NMPA Research Base of Regulatory Science for Medical Devices & Institute of Regulatory Science for Medical Devices, Huazhong University of Science and Technology, Wuhan 430074, Hubei, P. R. China.

NMPA Research Base of Regulatory Science for Medical Devices & Institute of Regulatory Science for Medical Devices, Huazhong University of Science and Technology, Wuhan 430074, Hubei, P. R. China.

Q.-R. Xu

The Key Laboratory of Tumor Molecular Diagnosis and Individualized Medicine of Zhejiang Province, Zhejiang Provincial People's Hospital, Affiliated People's Hospital, Hangzhou Medical College, Hangzhou 310014, Zhejiang, P. R. China.

E-mail address: xuqiuran@hmc.edu.cn (Q.-R. Xu)

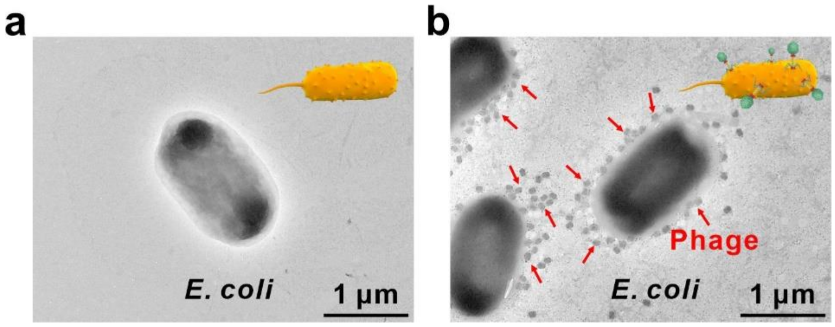

Figure S1. TEM images of *E. coli* BL21 incubation with T4 phage and schematic diagram of phage infection of *E. coli* (inset).

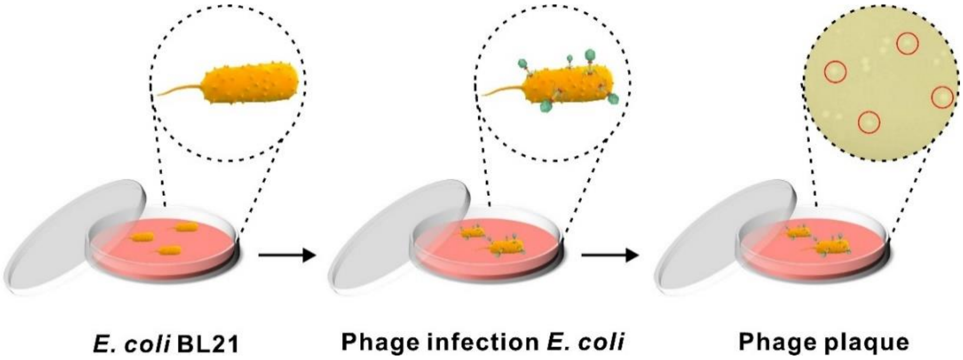

Figure S2. Formation of phage plaques by the T4 phage infection *E. coli* BL21.

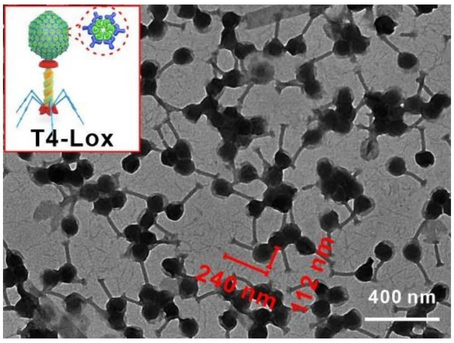

Figure S3. TEM image and corresponding schematic diagram of TL.

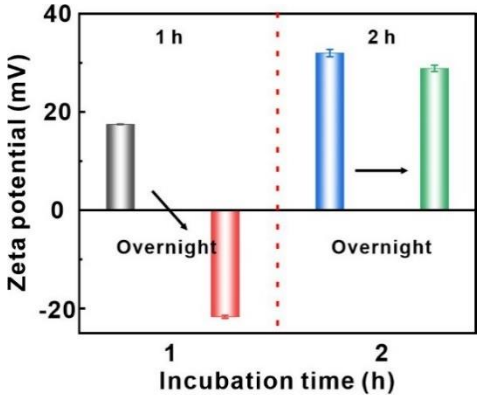

Figure S4. Zeta potential results after the incubation between poly-L-Lysine and TL at different times. Data are presented as the means ± s.d. (n = 4).

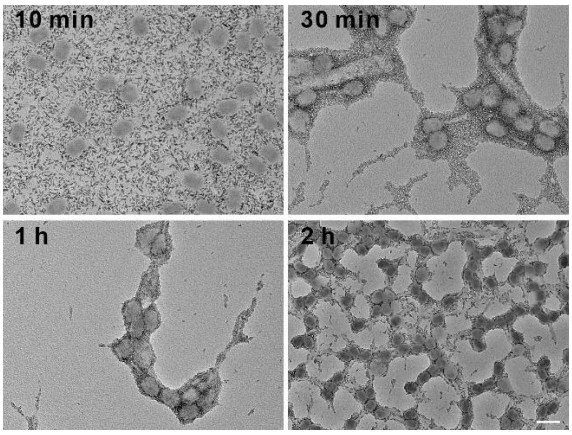

Figure S5. TEM images of the reaction of dextran iron (0.5 mg/mL) with TLD at different times (Scale bar: 200 nm).

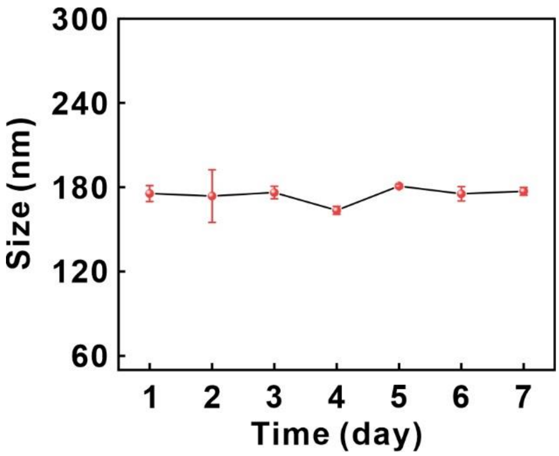

Figure S6. The hydrodynamic diameter of the TLDF at 4 ℃. Data are presented as means ± s.d. (n=3).

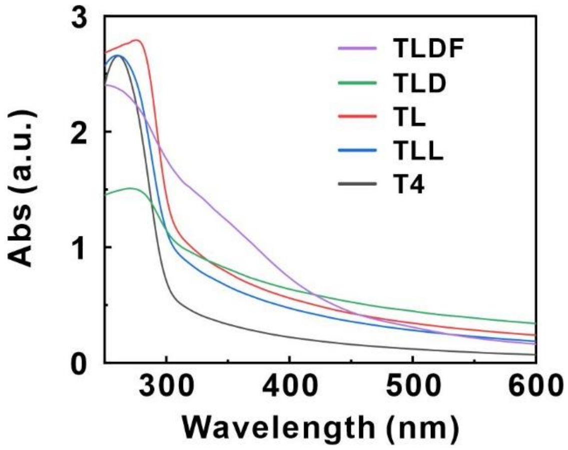

Figure S7. UV-vis absorption spectra of T4, TL, TLL, TLD, and TLDF probes.

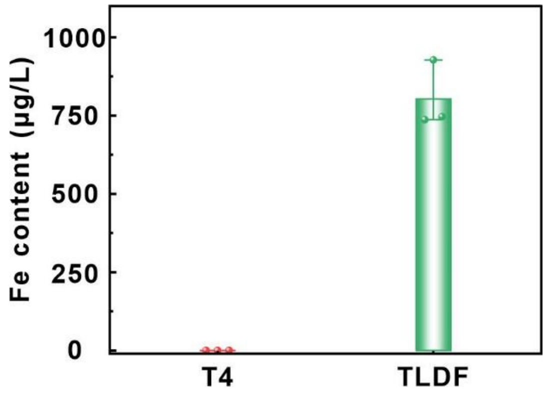

Figure S8. The amount of iron in the TLDF measurement by atomic absorption spectrophotometry. Data are presented as the means ± s.d. (n = 3).

a

Soc-Lox nucleotide sequences

GGTGGCTATGTTAATATTAAGACCTTTACCCATCCGGCAGGTGAAGGTAAAGAAGTGAAAGGCATGGAAGTTAGTGTTCCGT
TTGAAATCTATAGCAATGAACATCGCATTGCCGATGCACATTATCAGACCTTTCCGAGCGAAAAAGCAGCCTATACCACCGTT
GTGACCGATGCCGCAGATTGGCGTACCAAAAATGCAGCAATGTTTACCCCGACCCCGGTGAGCGGCCAAGCTTCTGCCATG
AACAACAACGATATCGAATACAACGCCCCGAGTGAAATTAAGTATATTGATGTGGTTAACACCTACGATCTGGAAGAAGAAG
CCAGTAAAGTTGTTCCGCATGGCGGCTTTAATTATATTGCAGGCGCCAGCGGTGACGAATGGACCAAACGTGCCAATGATCG
TGCATGGAAACATAAACTGCTGTATCCGCGTCTGGCCCAGGATGTTGAAGCCCCGGATACCAGCACCGAAATTCTGGGTCAT
AAAATTAAGGCCCCGTTTATTATGGCCCCGATTGCAGCCCATGGCCTGGCACATACCACCAAAGAAGCCGGCACCGCCCGC
GCAGTGAGCGAATTTGGTACCATTATGAGTATTAGCGCATATAGCGGCGCCACCTTTGAAGAAATTAGTGAAGGTCTGAATG
GCGGTCCGCGTTGGTTTCAGATCTATATGGCAAAAGATGATCAGCAGAATCGCGATATTCTGGATGAAGCCAAAAGCGATGG
TGCAACCGCAATTATTCTGACCGCAGATAGTACCGTGAGTGGTAATCGTGATCGTGATGTGAAAAATAAGTTTGTGTATCCGT
TTGGCATGCCGATTGTTCAGCGCTATCTGCGTGGTACCGCAGAAGGTATGAGTCTGAATAATATCTATGGTGCAAGCAAACA
GAAAATTAGTCCGCGTGATATTGAAGAAATTGCCGGTCATAGCGGCCTGCCGGTGTTTGTGAAAGGTATTCAGCATCCGGAA
GATGCCGATATGGCCATTAAGCGTGGCGCAAGTGGTATTTGGGTGAGCAATCATGGTGCACGCCAGCTGTATGAAGCACCG
GGTAGCTTTGATACCCTGCCGGCAATTGCCGAACGCGTTAATAAGCGTGTTCCGATTGTTTTTGATAGCGGTGTTCGCCGTG
GTGAACATGTGGCAAAAGCCCTGGCCAGTGGCGCAGATGTTGTGGCACTGGGCCGTCCGGTTCTGTTTGGTCTGGCCCTG
GGTGGTTGGCAGGGCGCATATAGTGTTCTGGATTATTTTCAGAAAGACCTGACCCGCGTTATGCAGCTGACCGGCAGCCAG
AATGTTGAAGATCTGAAAGGTCTGGATCTGTTTGATAATCCGTATGGTTATGAATATTAA

b

A portion of DNA sequencing results

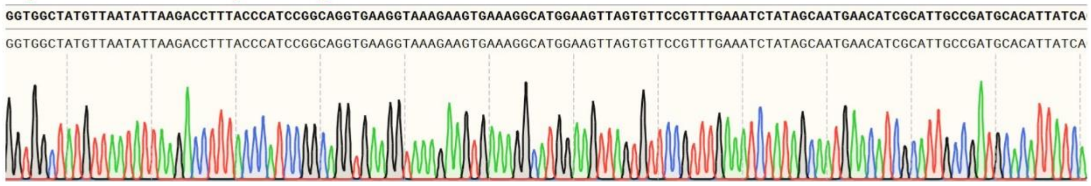

Figure S9. (**a**) The Soc-Lox nucleotide sequence (stop codon marked in red). (**b**) A portion of DNA sequencing results using T7 terminator primer.

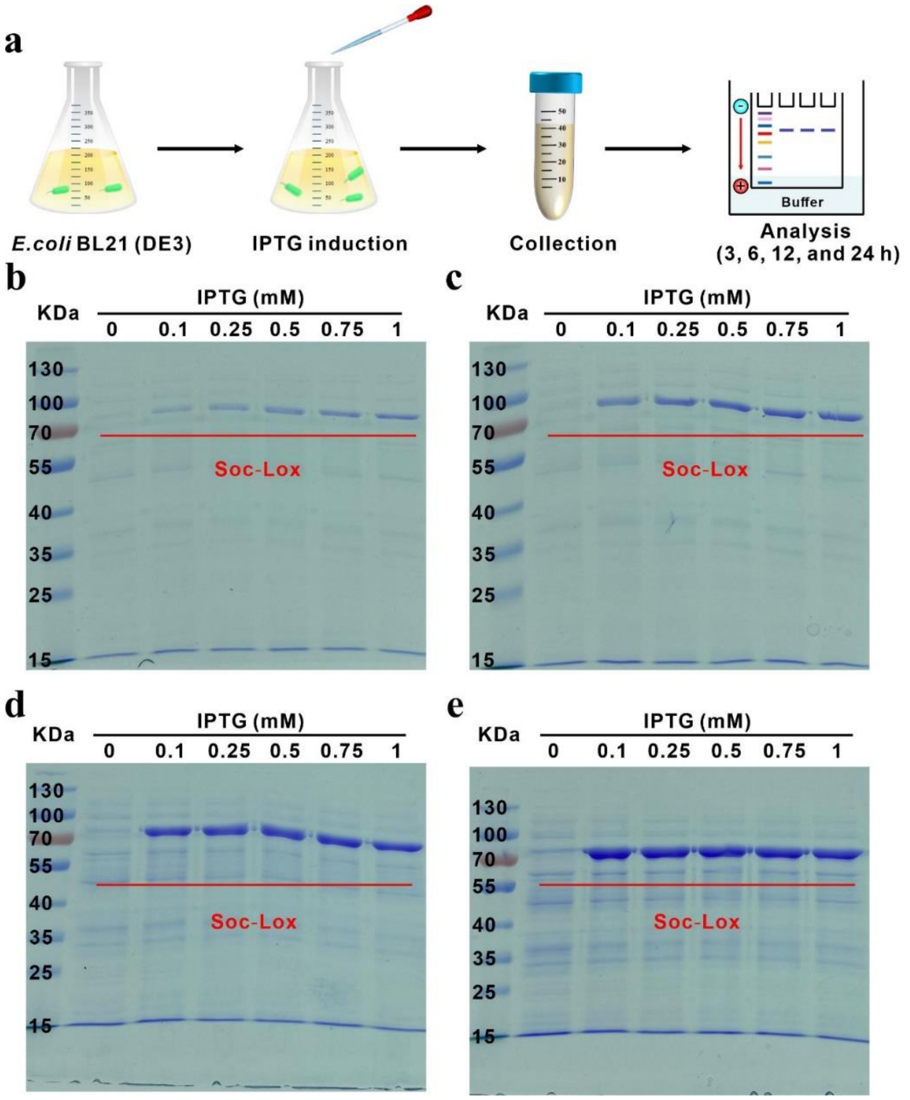

Figure S10. (**a**) Schematic diagram of Soc-Lox protein expression. SDS-PAGE results of protein expression induction by different concentrations of IPTG at 3 h (**b**), 6 h (**c**), 12 h (**d**), and 24 h (**e**).

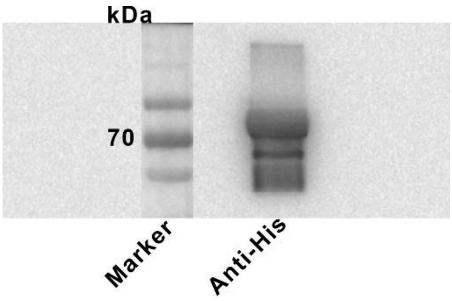

Figure S11. WB detection the Soc-Lox protein using His-tag antibody.

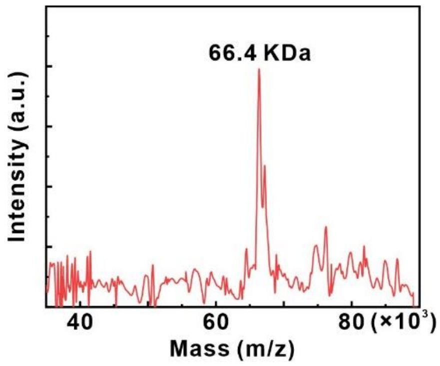

Figure S12. Mass Spectrometry (AB Sciex 5800 MALDI-TOF/TOF^{TM}) characterization of the Soc-Lox protein.

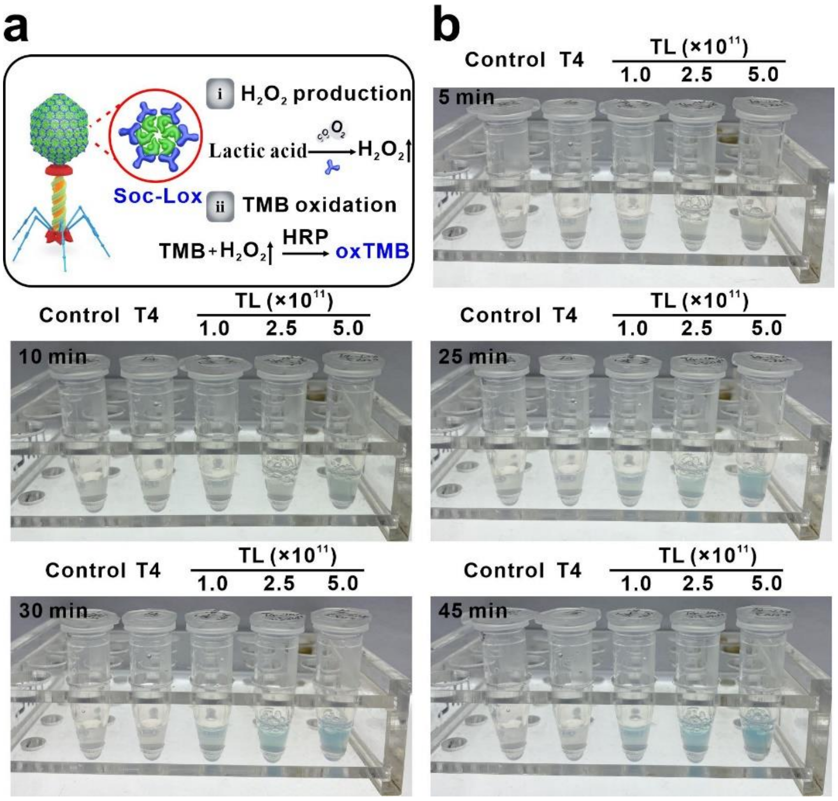

Figure S13. (**a**) Schematic illustration of oxTMB production in a cascade-like manner. (**b**) The production of H_2O_2 by different concentrations of TL at 5, 10, 25, 30, and 45 min.

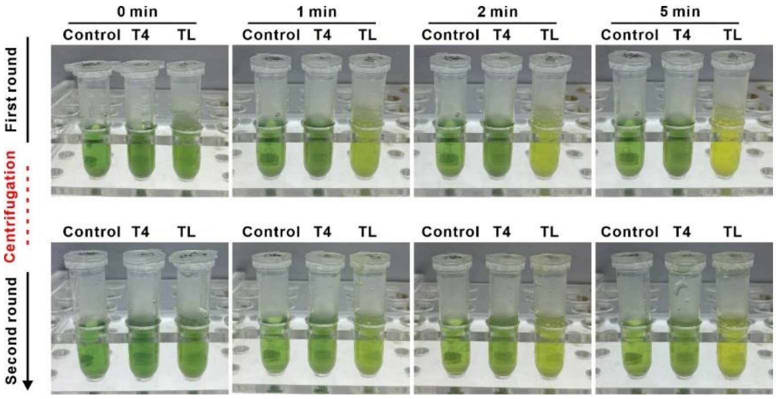

Figure S14. The color change of 2,6-dichloroindophenol during T4 and TL catalyst lactic acid reaction.

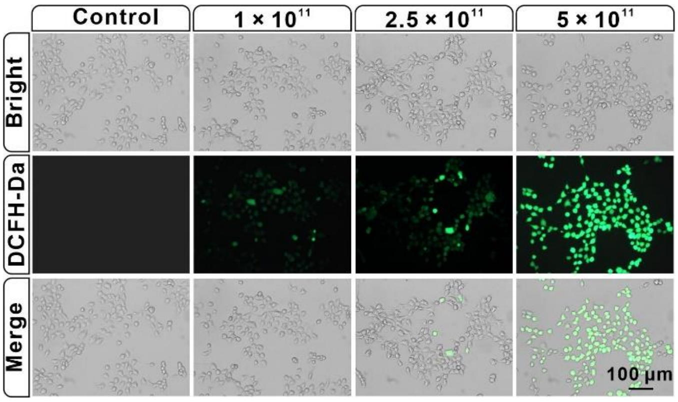

Figure S15. DCFH-Da fluorescence signals in the cells after treatment with different concentrations of TL.

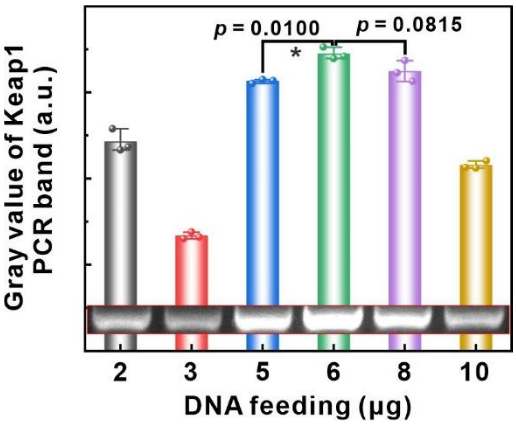

Figure S16. PCR Amplification bands of Keap1 nucleic acid (inset) and corresponding quantitative results. Data are presented as the means ± s.d. (n = 3). Statistical differences were calculated using a two-tailed Student’s t-test, *: $p < 0.05$.

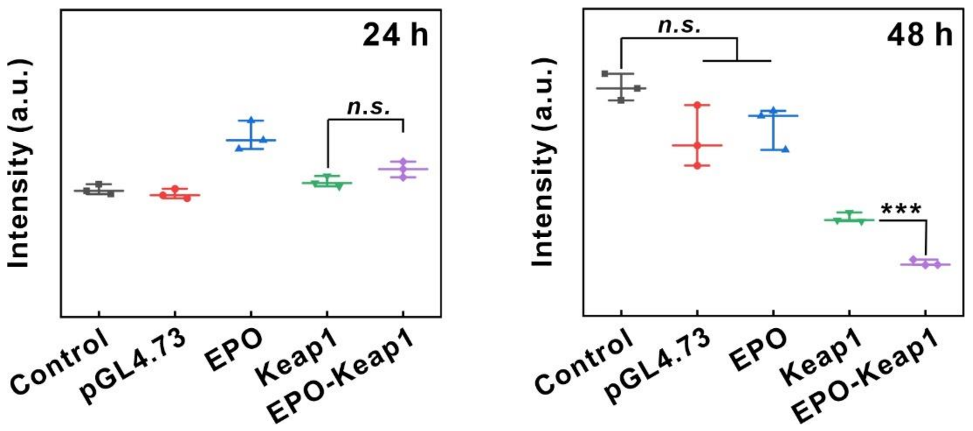

Figure S17. Grayscale quantitative of Nrf2 protein degradation at 24 h or 48 h in an anoxic environment. Data are presented as the means ± s.d. (n = 3). Statistical differences were calculated using a two-tailed Student's t-test, ***: $p < 0.001$ and *n.s.* means no statistically significant difference.

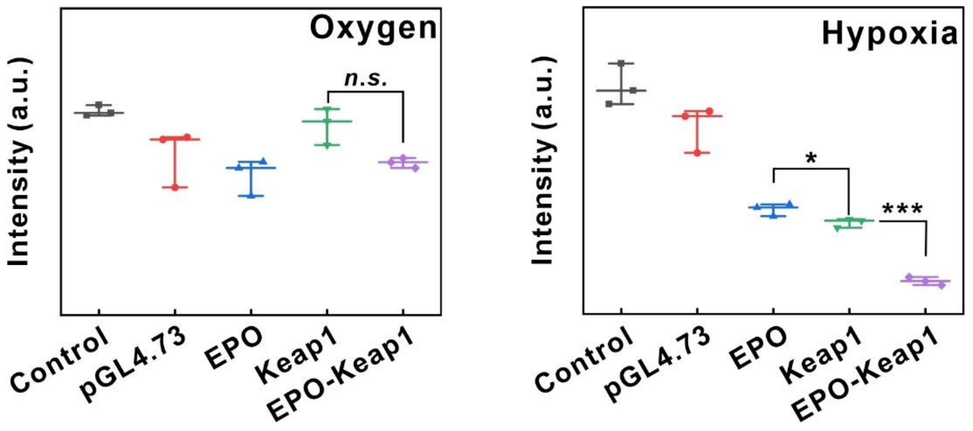

Figure S18. Grayscale quantitative of Nrf2 protein degradation at normoxia or hypoxia environment. Data are presented as means ± s.d. (n=3). Statistical difference was calculated using a two-tailed student's t-test, *: $p<0.05$, ***: $p<0.001$, and *n.s.* means no statistically significant difference.

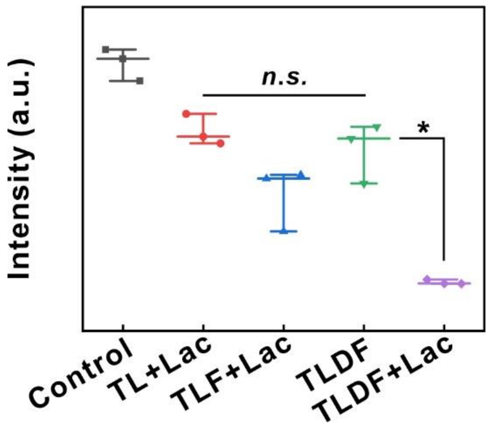

Figure S19. Grayscale quantitative of Nrf2 protein after different treatments. Data are presented as means ± s.d. (n=3). Statistical difference was calculated using a two-tailed student's t-test, *: $p<0.05$ and *n.s.* means no statistically significant difference.

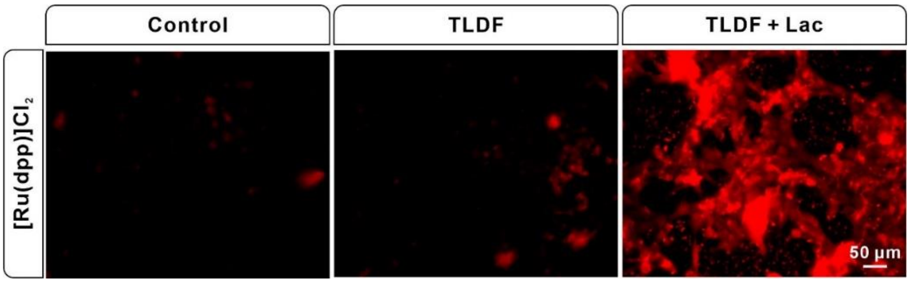

Figure S20. Changes in intracellular oxygen content during the reaction of TLDF with lactic acid detected by ruthenium dichloride.

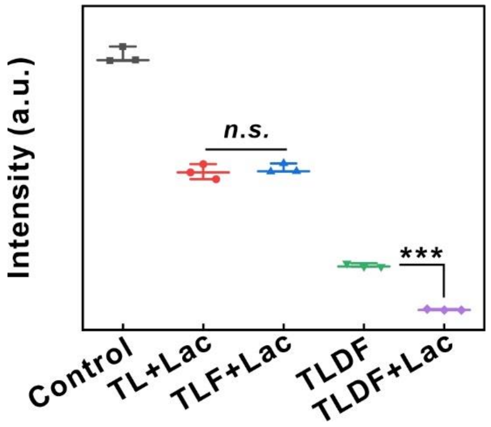

Figure S21. Grayscale quantitative of HO-1 protein after different treatments. Data are presented as means ± s.d. (n=3). Statistical difference was calculated using a two-tailed student's t-test, ***: $p<0.001$ and *n.s.* means no statistically significant difference.

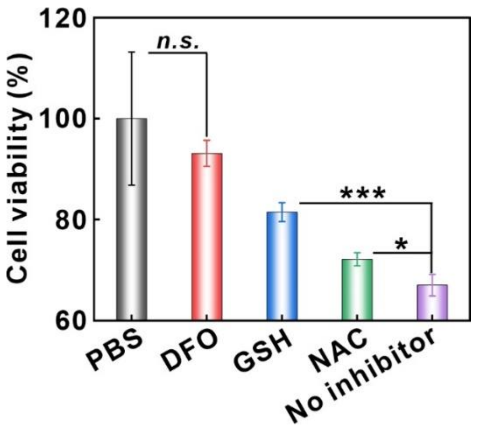

Figure S22. Survival of 4T1 cells after the addition of different ferroptosis inhibitors, including deferoxamine mesylate (800 μM), GSH (2.5 mM), and N-acetyl-L-cysteine (5 mM) (n = 3 independent samples). Data are presented as means ± s.d. (n=3). Statistical difference was calculated using a two-tailed student's t-test, *: p<0.05, ***: p<0.001, and *n.s.* means no statistically significant difference.

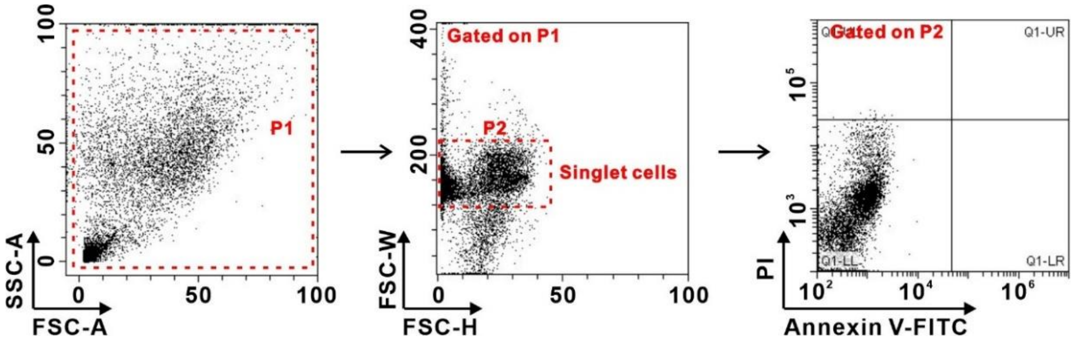

Figure S23. The gating strategy for apoptosis experiments.

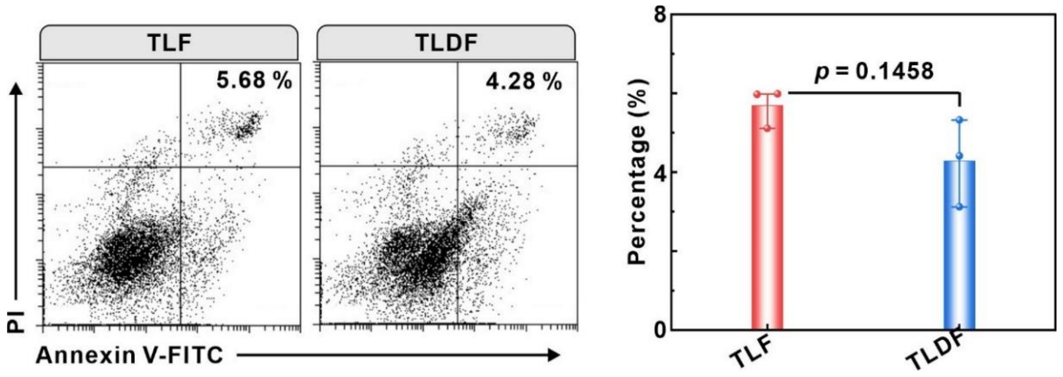

Figure S24. Flow cytometry detection apoptosis cell induced by different probes. Data are presented as the means ± s.d. (n = 3). Statistical differences were calculated using a two-tailed Student's t-test, *: $p < 0.05$.

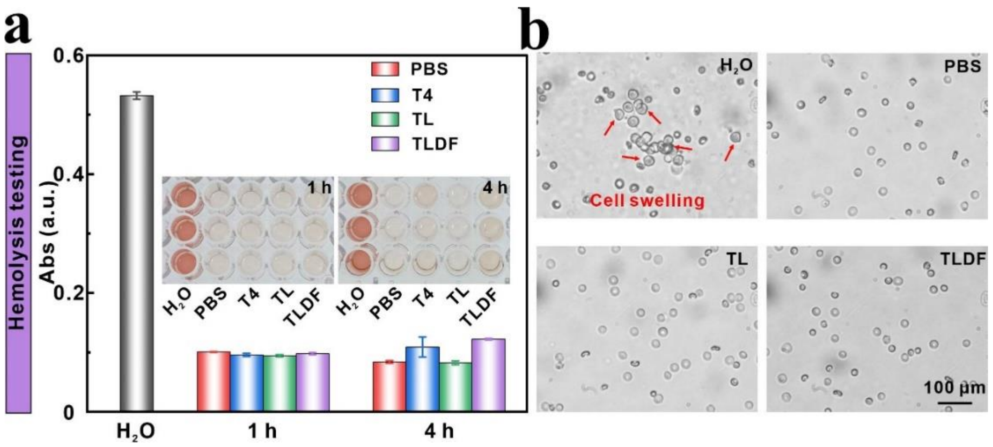

Figure S25. Hemolysis testing after mouse erythrocytes incubation with the TLDF for 1 h or 4 h. The content of hemoglobin (**a**) and microscope images of red blood cells (**b**). Error bars indicate SD.

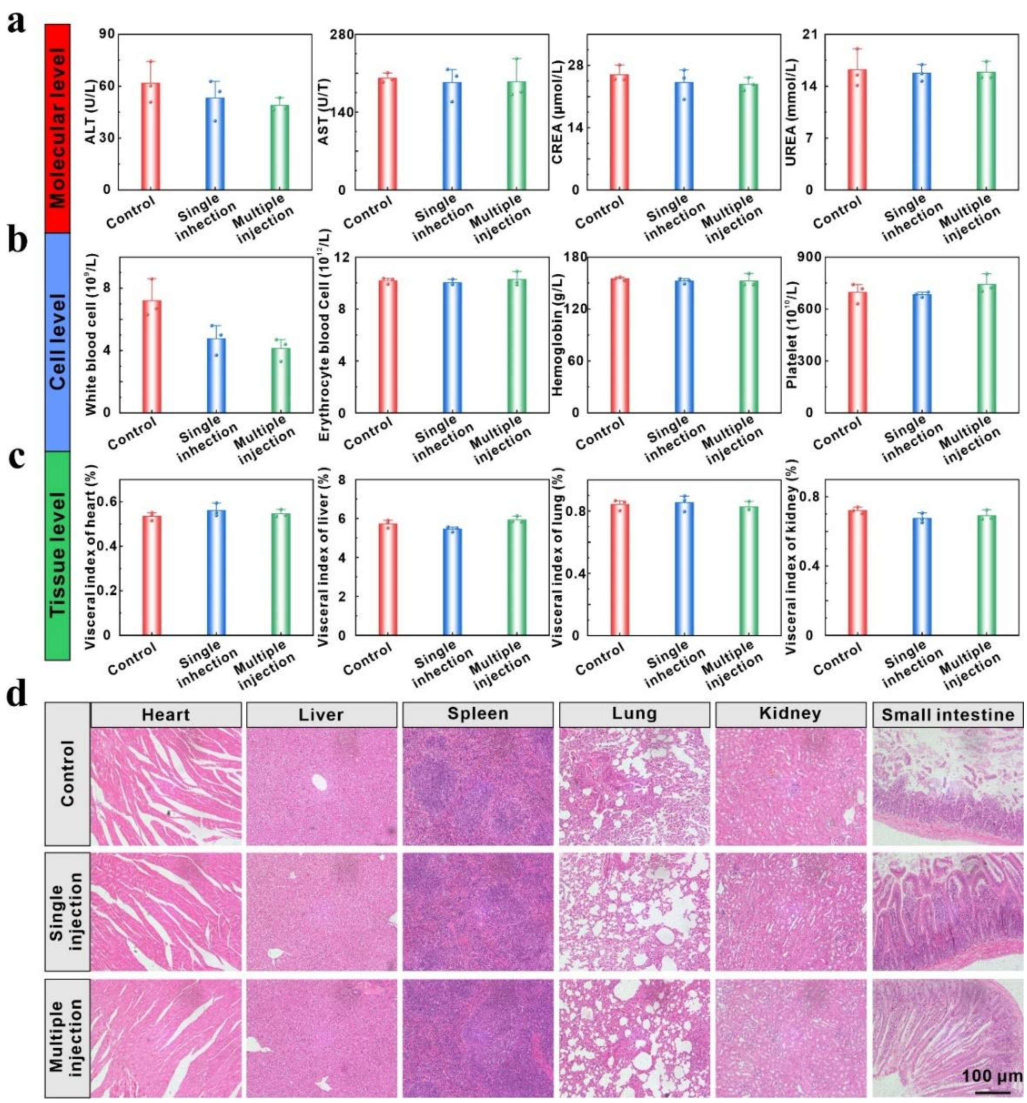

Figure S26. **The biocompatibility of TLDF *in vivo***. (**a**) AST (U/L), Urea (mmol/L), Crea (μmol/L), and ALT (U/L) in healthy mice after different treatments. Data are presented as the means ± s.d. (n = 3). (**b**) White blood cell, erythrocyte blood cell, hemoglobin, and platelet in healthy mice after different treatments. Data are presented as the means ± s.d. (n = 3). (**c**) Visceral index in healthy mice after different treatments. Data are presented as the means ± s.d. (n = 3). (**d**) H&E staining of the heart, liver, spleen, lung, and kidney of mice after different treatments.

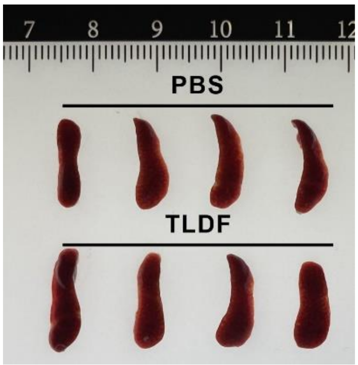

Figure S27. Pictures of the spleen after treatment with the TLDF probe.

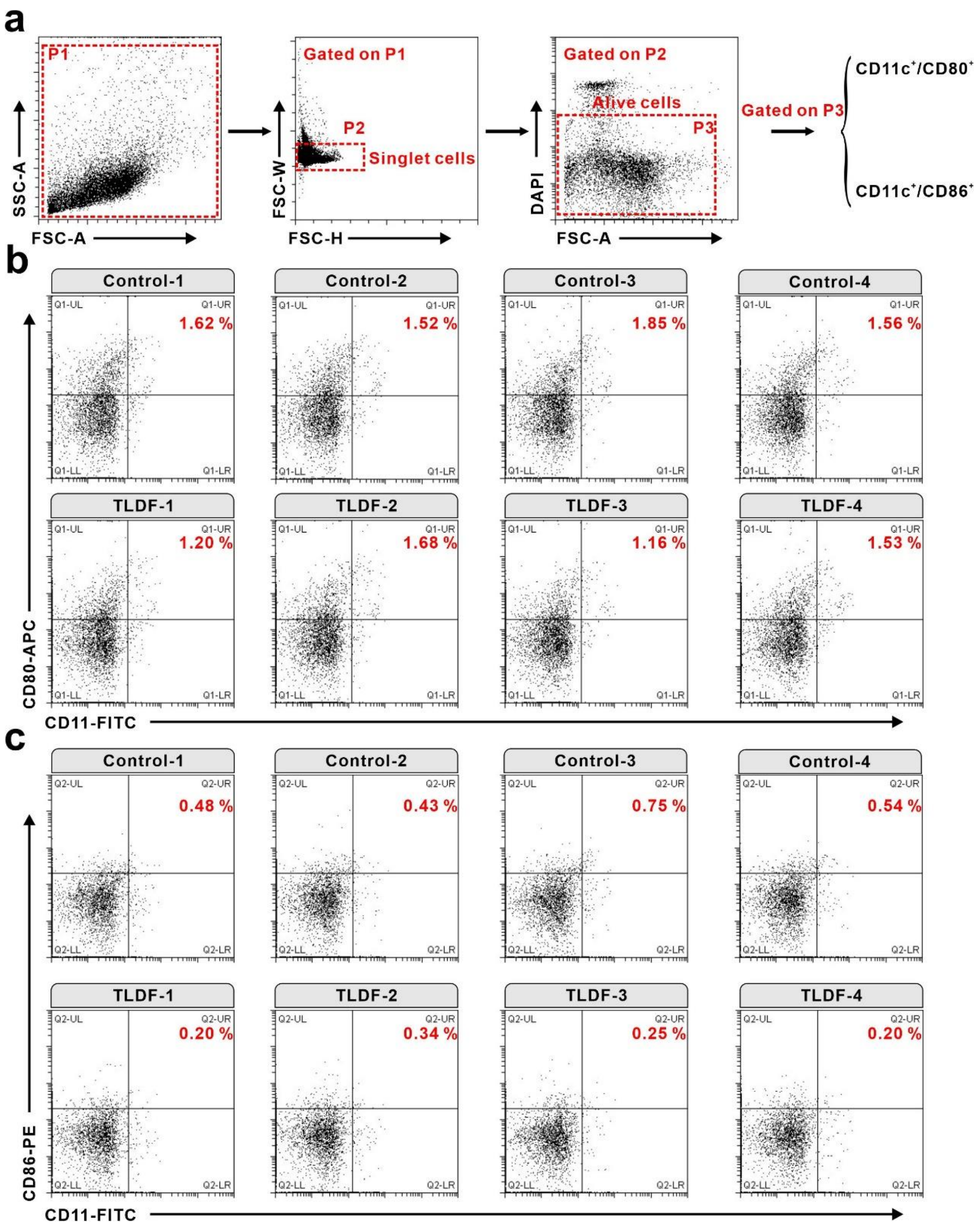

Figure S28. (**a**) Representative gating strategy of flow cytometry analysis. (**b**), (**c**) Representative flow cytometry analysis of the population of DC maturation in the spleen after treatment with TLDF on day 30 (n = 4).

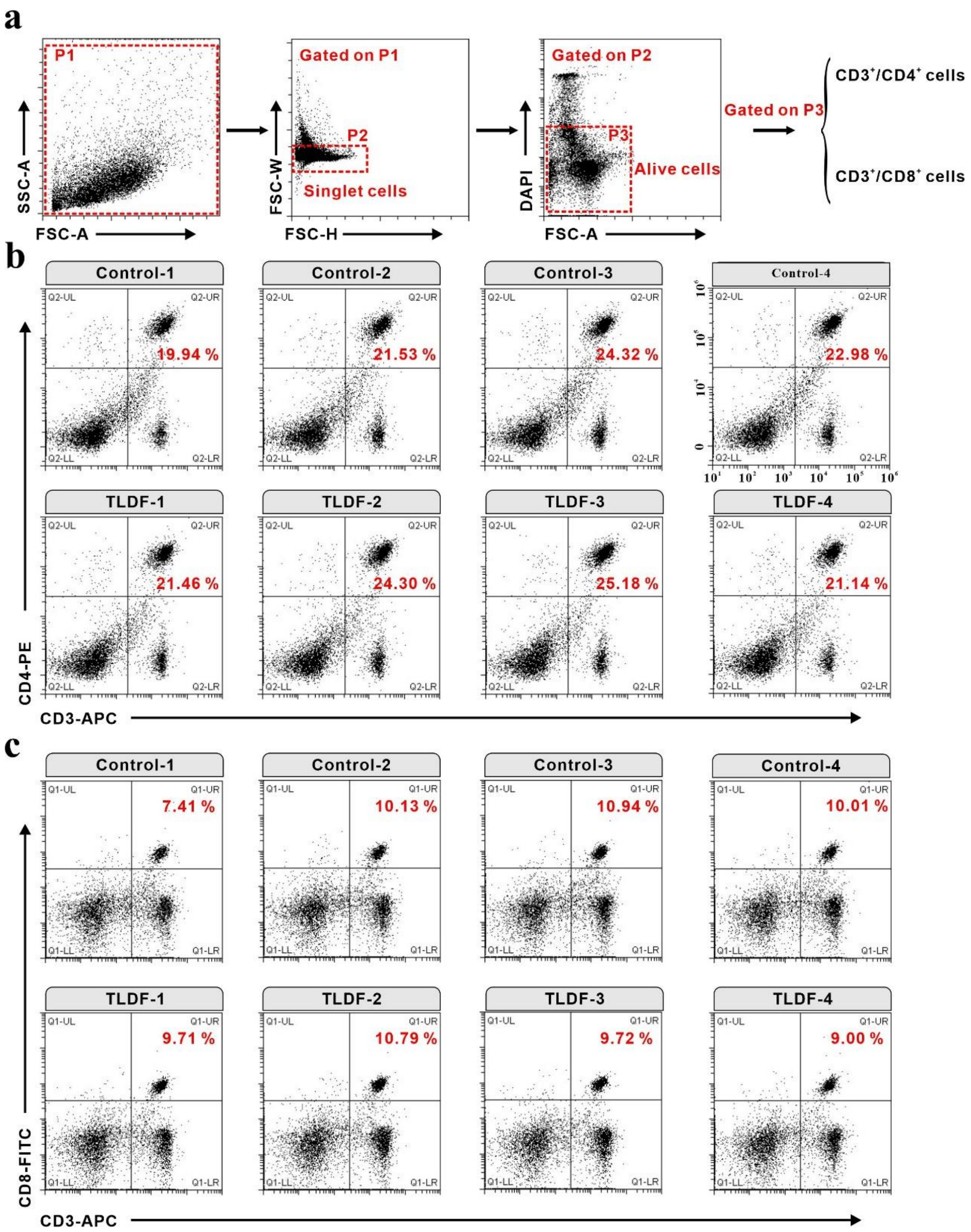

Figure S29. (**a**) Representative gating strategy of flow cytometry analysis. (**b**), (**c**) Representative flow cytometry analysis of the population of T cells maturation in the spleen after treatment with TLDF on day 30 (n = 4).

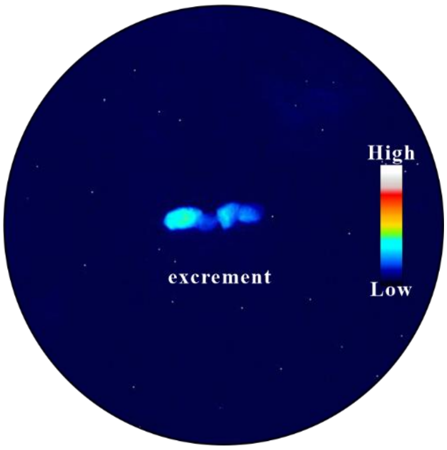

Figure S30. Fluorescence image of mouse excrement at 8 h after treatment with the TLDF@C.

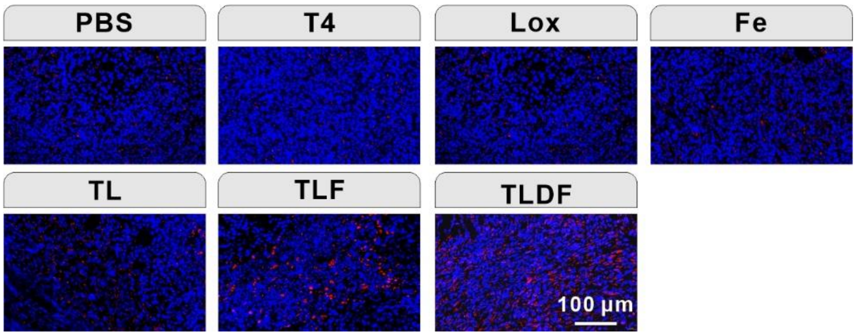

Figure S31. The immunofluorescence staining of HIF-1α at the tumor site.

Table S1. The valence state, binding energy (BE), and the percentage of different valence states of iron in TLDF probes. BE binding energy (nm).

Name	Peak BE	Atomic %
Fe^{2+} $2p_{3/2}$	709.14	26.03
Fe^{2+} $2p_{1/2}$	722.18	
Fe^{3+} $2p_{3/2}$	710.88	73.97
Fe^{3+} $2p_{1/2}$	724.28	
Fe^{2+} $2p_{3/2}$ satellite peak	714.21	/
Fe^{3+} $2p_{3/2}$ satellite peak	718.99	
Fe^{2+} $2p_{1/2}$ satellite peak	727.87	/
Fe^{3+} $2p_{1/2}$ satellite peak	732.99	

Table S2. Primer sequence of different experiments.

Gene		Primer
Soc-Lox	Forward	5’-TCCCGACTCTGCTGCTGTTCA-3’
	Reverse	5’-TGCTAGTTATTGCTCAGCGG-3’
Keap1	Forward	5’-CTTATGCAGCCCGAACCCAAG-3’
	Reverse	5’-CTAGATTATCAGCATGTGCAATTCT-3’
RV primer 3	Forward	5’-CTAGCAAAATAGGCTGTCCC-3’
RV primer 4	Reverse	5’-GACGATAGTCATGCCCCGCG-3’

Table S3. Routine blood testing in healthy mice after treatment with the TLDF.

Parameter	PBS	Single injection	Multiple injection	Reference range	Unit
WBC	7.20±1.23	4.77±0.97	4.13±0.74	0.80-10.60	10^9/L
Lymph	5.67±1.34	3.70±0.85	3.10±0.36	0.60-8.90	10^9/L
Mon	0.20±0.00	0.10±0.00	0.13±0.12	0.04-1.40	10^9/L
Gran	1.33±0.12	0.97±0.15	0.90±0.26	0.23-3.60	10^9/L
Lymph%	78.37±4.67	76.80±2.70	75.63±4.22	40.00-92.00	%
Mon%	2.37±0.35	2.50±0.30	3.47±0.87	0.90-18.00	%
Gran%	19.27±4.38	20.70±2.43	20.90±3.40	6.50-50.00	%
RBC	11.07±0.55	10.18±0.24	10.27±0.55	6.50-11.50	10^{12}/L
HCT	53.30±2.15	48.63±1.36	47.40±2.43	35.00-55.00	%
MCV	48.20 ±0.46	47.83±0.25	46.20±0.56	44.00-55.00	fL
MCH	15.47±0.40	15.17±0.21	14.80±0.17	13.00-18.00	pg
RDW	15.40±0.17	16.20±0.17	16.53±0.57	12.00-19.00	%
MPV	5.47±0.21	5.77±0.23	5.63±0.51	3.80-6.00	fL
HGB	172.00±7.81	155.00±2.00	152.33±7.51	110-180	g/L
MCHC	322.00±7.81	318.00±5.20	321.00±1.00	300-360	g/L
PLT	696.00±58.40	683.33±14.57	745.67±49.90	400-1600	10^9/L
